# Hidden Optical Nonlinearities in Linear Spectra of Quantum Emitter Arrays

**DOI:** 10.1002/nap2.70252

**Published:** 2026-08-19

**Authors:** Sricharan Raghavan‐Chitra, Arghadip Koner, Joel Yuen‐Zhou

**Affiliations:** ^1^ Department of Chemistry and Biochemistry University of California San Diego La Jolla California USA

## Abstract

Classical optical frameworks such as the discrete dipole approximation (DDA) assume that the linear spectrum of coupled quantum emitters can be computed solely from the linear susceptibilities of individual constituents. However, recent polariton studies show that cavity linear response can encode nonlinear optical susceptibilities. Here, we demonstrate that this phenomenon is more general: emitter–emitter interactions allow nonlinearities of individual emitters to emerge in the linear response of arrays, without cavities or permutational symmetry. To illustrate this phenomenon, we show linear spectra for coupled heterodimers and linear chains, and demonstrate that Raman features of individual monomers show up as vibrational sidebands of collective resonances. Moreover, tuning Raman‐type anharmonicities enables systematic control of spectral features, establishing a genuine quantum optical effect in molecular aggregates and quantum emitter arrays, which goes beyond mean‐field descriptions in light‐matter interactions.

## Graphical abstract

Exact quantum calculations reveal Raman‐like vibrational signatures in the linear absorption spectrum of a coupled chlorophyll dimer that are absent from classical optical descriptions. These features occur at energy separations set by the monomer vibrational modes, showing how intermolecular coupling exposes hidden nonlinear photophysical processes within an otherwise linear optical response.

## 1 Introduction

The optical response of quantum emitter arrays [1, 2], ranging from molecular aggregates [3, 4, 5, 6, 7, 8, 9, 10, 11, 12] to solid‐state [13] and cavity‐coupled platforms [14, 15], lies at the heart of nanophotonics [16, 17] and quantum optics [18], with implications for light harvesting [19, 20], sensing [21, 22, 23], and quantum technologies [24, 25, 26]. A central objective in these settings is to understand how microscopic photophysical processes are encoded in macroscopic observables, particularly in linear optical spectra, which are routinely used to infer structure and dynamics. Traditionally, such spectra are interpreted as probes of single‐excitation physics [27], providing access to transition energies, oscillator strengths, and coherence properties, while higher‐order processes, such as Raman scattering and multiphoton interactions, are presumed to belong exclusively to the domain of nonlinear spectroscopy [27]. This viewpoint is closely tied to a central tenet of classical linear optics [28]: the macroscopic response of a composite system is fully determined by the linear susceptibility $\chi^{\left(1\right)}\left(\omega \right)$ of its individual constituents. This assumption, in turn, underlies a broad class of mean‐field frameworks, including the discrete dipole approximation (DDA) [29, 30, 31], the coherent potential approximation (CPA) [32, 33], and the coherent exciton scattering (CES) approximation [34]. This paradigm has proven remarkably fruitful, underpinning our understanding of electromagnetic scattering by arbitrarily shaped particles [35], the properties of disordered alloys [32, 36, 37], and the optical properties of quantum emitter arrays [29, 30, 38, 39].

However, recent studies of light‐matter systems [40, 41] have called this classical picture into question by showing that the cavity‐modified linear response of a molecular ensemble can encode contributions from nonlinear susceptibilities, specifically Raman‐type contributions conventionally regarded as components of $\chi^{\left(3\right)}\left(\omega \right)$ and therefore, by the textbook account, invisible to linear spectroscopy. Two ingredients underlie this counterintuitive result: the permutational symmetry of identical molecules coupled to a common cavity mode [42, 43], and the nature of the photophysical processes in the emitters that contribute to the overall linear response of the system. Subsequent work [44] demonstrated that permutational symmetry alone, even in the absence of a cavity, is sufficient to generate analogous corrections in molecular aggregates, while simultaneously clarifying the precise regime in which CPA and CES are exact and where they break down. However, such systems are highly idealized and rarely realized in large molecular ensembles. Consequently, the scope of these results remains intrinsically tied to the presence of strict many‐body permutational symmetry [45, 46], thereby limiting their applicability to more general structurally disordered emitter arrays.

In this article, we demonstrate that this phenomenon is far more general: emitter–emitter interactions enable intrinsic nonlinearities of individual emitters to manifest within the linear response of arrays, without requiring cavities or permutational symmetry. We illustrate this mechanism using the simplest nonsymmetric coupled system: a heterodimer. Despite its minimal structure, this model captures essential physics relevant to a wide range of contemporary platforms, including coupled NV centers [47], lanthanide dimers [48], quantum emitters in two‐dimensional materials [49], and molecular aggregates [50]. Although its linear response is routinely obtained via exact diagonalization or matrix inversion, we instead derive it through a Dyson expansion [27], treating inter‐emitter coupling as the perturbation. This framework reveals the underlying photophysical processes governing the heterodimer's linear response. By mapping the dynamics onto ladder diagrams [51], we show that Raman‐type processes contribute to the array's linear response, akin to phenomena in polaritonic and permutationally symmetric aggregates. For the Chl522–Chl520 heterodimer [50], Raman signatures of one emitter appear as sidebands to the absorption peak of the other, enabling extraction of Raman information from linear spectra. Extending this framework to linearly coupled quantum emitters, we identify regimes where such features were previously overlooked. Controlled tuning of Raman‐type anharmonicities thus provides systematic control over spectral features, revealing a genuine quantum optical effect long sought in the field of molecular aggregates [52, 53, 54].

The article is organized as follows. In Section 2, we introduce the heterodimer Hamiltonian and derive its linear response by resumming terms in the Dyson expansion, with the detailed derivation presented in Supporting Information S1: Section 1. Using these expressions, we simulate the linear spectrum of the Chl522–Chl520 heterodimer within a minimal three‐level basis sufficient to capture the phenomenon of interest. In Section 3, we extend the analysis to more physically relevant linear arrays of quantum emitters. Using parameter regimes previously explored in the molecular‐aggregate literature, we identify overlooked Raman features in the linear response of J‐aggregates. Our results show that aggregate linear absorption spectra inherently encode higher‐order nonlinear susceptibilities, thereby enriching the structure–spectra relationship and revealing a chemically tunable handle for the rational design of quantum emitter arrays.

## 2 Linear Response of Heterodimer

In this section, we derive the linear absorption spectrum of an arbitrary heterodimer comprising monomers A and B. The linear absorption spectrum of a system driven by a weak incident laser field is given by the following equation [27]:

(1) $$\sigma \left(\omega \right)\propto -I\langle \hat{\mu }\;\hat{G}\left(\omega \right)\;\hat{\mu }\rangle ,$$

where $\hat{\mu }$ is the transition dipole moment operator coupling the system to the electric field of the incoming laser, and $\hat{G}\left(\omega \right)=\frac{1}{\omega -\hat{H}+i0^{+}}$ is the retarded Green's function of the system, with $\hat{H}$ denoting the total Hamiltonian. The expectation value 〈⋅〉 is taken with respect to the initial state of the system (typically, the ground state; see Equation 8). For a collection of heterodimers, the total dipole operator decomposes as $\hat{\mu }={\hat{\mu }}_{A}+{\hat{\mu }}_{B}$, and the full Hamiltonian takes the following form:

(2) $$\hat{H}={\hat{H}}_{A}+{\hat{H}}_{B}+{\hat{H}}_{I}.$$

Under the Born–Oppenheimer approximation, the molecular Hamiltonian of monomer A or B is given by the following equation:

(3) $${\hat{H}}_{A/B}={\hat{T}}_{A/B}+V_{g}\left(q_{A/B}\right)\;|g_{A/B}\rangle \langle g_{A/B}|+V_{e}\left(q_{A/B}\right)\;|e_{A/B}\rangle \langle e_{A/B}|,$$

where $\hat{T}$ is the nuclear kinetic energy operator, $V_{g/e}$ denote the ground and excited potential energy surfaces (PESs), and $q_{A/B}$ represents the set of intramolecular vibrational coordinates of monomer A or B. The interaction Hamiltonian captures the excitonic coupling between the two monomers [3]:

(4) $${\hat{H}}_{I}=J\;|e_{A}\rangle \langle e_{B}|+h.c.,$$

where J is the excitonic coupling strength between the locally excited states of A and B.

To make the derivation tractable while retaining the essential physics, we employ a reduced basis in which each monomer is represented as a three‐level (or Λ‐type) system: two vibrational levels on the ground‐state PES and one vibrational level on the excited‐state PES. For simplicity, all Franck–Condon overlaps between vibronic levels are set to unity. Although this truncation simplifies the algebra considerably, it captures the photophysical behavior of interest, and the results can be straightforwardly extended to an arbitrary vibronic basis. Within this reduced basis, the monomer Hamiltonians take the following explicit form:

(5) $$\begin{array}{ll} {\hat{H}}_{A} & =\omega_{g,0,A}\;|g_{A},0_{A}\rangle \langle g_{A},0_{A}|\\ & \;+\omega_{g,1,A}\;|g_{A},1_{A}\rangle \langle g_{A},1_{A}|+\omega_{e,0,A}\;|e_{A},0_{A}^{\prime }\rangle \langle e_{A},0_{A}^{\prime }|, \end{array}$$

(6) $$\begin{array}{ll} {\hat{H}}_{B} & =\omega_{g,0,B}\;|g_{B},0_{B}\rangle \langle g_{B},0_{B}|\\ & \;+\omega_{g,1,B}\;|g_{B},1_{B}\rangle \langle g_{B},1_{B}|+\omega_{e,0,B}\;|e_{B},0_{B}^{\prime }\rangle \langle e_{B},0_{B}^{\prime }|, \end{array}$$

(7) $$\begin{array}{ll} {\hat{H}}_{I} & =J\;|e_{A};g_{B}\rangle \langle g_{A};e_{B}|+h.c.. \end{array}$$

here $\omega_{g,0,A\left(B\right)}$ is the energy of the global ground state $|g_{A\left(B\right)},0_{A\left(B\right)}\rangle$ of monomer A(B), whereas $\omega_{g,1,A\left(B\right)}$ and $\omega_{e,0,A\left(B\right)}$ denote the energies of the vibrationally excited level on the ground‐state PES and the vibrational ground level on the excited‐state PES, respectively. The coupling J mediates energy transfer between the local excitations $|e_{A};g_{B}\rangle$ and $|g_{A};e_{B}\rangle$, where the semicolon denotes a tensor product (i.e., $|e_{A};g_{B}\rangle \equiv |e_{A}\rangle ⊗|g_{B}\rangle$). At zero temperature, the linear absorption of this heterodimer system under a perturbative light source is given by the following equation:

(8) $$\sigma \left(\omega \right)\propto -I\;\langle g_{A},0_{A};g_{B},0_{B}|\;\hat{\mu }\;\hat{G}\left(\omega \right)\;\hat{\mu }\;|g_{A},0_{A};g_{B},0_{B}\rangle ,$$

where the total dipole operator is $\hat{\mu }={\hat{\mu }}_{A}+{\hat{\mu }}_{B}$, with: ${\hat{\mu }}_{A}=\mu_{M}^{A}\;|g_{A}\rangle \langle e_{A}|+h.c.$, ${\hat{\mu }}_{B}=\mu_{M}^{B}\;|g_{B}\rangle \langle e_{B}|+h.c.,$ and $\mu_{M}^{A/B}$ is the transition dipole matrix element of monomer A/B. Expanding the expectation value yields four distinct contributions:

(9) $$\begin{array}{ll} & \langle g_{A},0_{A};g_{B},0_{B}|\;\hat{\mu }\;\hat{G}\left(\omega \right)\;\hat{\mu }\;|g_{A},0_{A};g_{B},0_{B}\rangle \\ & =|\mu_{M}^{A}{|}^{2}\langle e_{A},0_{A}^{\prime };g_{B},0_{B}|\;\hat{G}\left(\omega \right)\;|e_{A},0_{A}^{\prime };g_{B},0_{B}\rangle \\ & \;+|\mu_{M}^{A}\|\mu_{M}^{B}|\langle e_{A},0_{A}^{\prime };g_{B},0_{B}|\;\hat{G}\left(\omega \right)\;|g_{A},0_{A};e_{B},0_{B}^{\prime }\rangle \\ & \;+|\mu_{M}^{A}\|\mu_{M}^{B}|\langle g_{A},0_{A};e_{B},0_{B}^{\prime }|\;\hat{G}\left(\omega \right)\;|e_{A},0_{A}^{\prime };g_{B},0_{B}\rangle \\ & \;+|\mu_{M}^{B}{|}^{2}\langle g_{A},0_{A};e_{B},0_{B}^{\prime }|\;\hat{G}\left(\omega \right)\;|g_{A},0_{A};e_{B},0_{B}^{\prime }\rangle . \end{array}$$

The first and fourth terms are diagonal matrix elements of the retarded Green's function, corresponding to the creation and destruction of the exciton in the same monomer. The second and third terms are the off‐diagonal matrix elements of the retarded Green's function, corresponding to the creation of an exciton in one monomer and its destruction in the other. These elements and their diagrammatic interpretations are computed explicitly in Supporting Information S1: Section 1. The Dyson expansion method offers a computational advantage over exact‐diagonalization approaches for calculating spectra. This is because spectral calculations do not require the complete eigenvalue and eigenstate information obtained through exact diagonalization. We summarize here the final expressions obtained for various terms in Equation (9):

(10) $$\begin{array}{ll} \langle e_{A},0_{A}^{\prime };g_{B},0_{B}|\hat{G}\left(\omega \right)|e_{A},0_{A}^{\prime };g_{B},0_{B}\rangle & =\frac{G_{e,A}\left[1-J^{2}G_{e,A,v,B}\left(G_{e,B}+G_{v,A,e,B}\right)\right]}{1-J^{2}\left(G_{e,A}+G_{e,A,v,B}\right)\left(G_{e,B}+G_{v,A,e,B}\right)}, \end{array}$$

(11) $$\begin{array}{ll} \langle g_{A},0_{A};e_{B},0_{B}^{\prime }|\hat{G}\left(\omega \right)|g_{A},0_{A};e_{B},0_{B}^{\prime }\rangle & =\frac{G_{e,B}\left[1-J^{2}G_{v,A,e,B}\left(G_{e,A}+G_{e,A,v,B}\right)\right]}{1-J^{2}\left(G_{e,B}+G_{v,A,e,B}\right)\left(G_{e,A}+G_{e,A,v,B}\right)}, \end{array}$$

(12) $$\begin{array}{ll} \langle e_{A},0_{A}^{\prime };g_{B},0_{B}|\hat{G}\left(\omega \right)|g_{A},0_{A};e_{B},0_{B}^{\prime }\rangle & =\langle g_{A},0_{A};e_{B},0_{B}^{\prime }|\hat{G}\left(\omega \right)|e_{A},0_{A}^{\prime };g_{B},0_{B}\rangle \\ & =\frac{JG_{e,A}G_{e,B}}{1-J^{2}\left(G_{e,A}+G_{e,A,v,B}\right)\left(G_{e,B}+G_{v,A,e,B}\right)}, \end{array}$$

where

(13) $$\begin{array}{ll} G_{e,A} & =\frac{1}{\omega -\left(\omega_{e,0,A}+\omega_{g,0,B}\right)+i\frac{\gamma_{e,A}}{2}}, \end{array}$$

(14) $$\begin{array}{ll} G_{e,B} & =\frac{1}{\omega -\left(\omega_{e,0,B}+\omega_{g,0,A}\right)+i\frac{\gamma_{e,B}}{2}}, \end{array}$$

(15) $$\begin{array}{ll} G_{v,A,e,B} & =\frac{1}{\omega -\left(\omega_{g,1,A}+\omega_{e,0,B}\right)+i\frac{\gamma_{v,A}+\gamma_{e,B}}{2}}, \end{array}$$

(16) $$\begin{array}{ll} G_{e,A,v,B} & =\frac{1}{\omega -\left(\omega_{e,0,A}+\omega_{g,1,B}\right)+i\frac{\gamma_{e,A}+\gamma_{v,B}}{2}}. \end{array}$$

here $\gamma_{e,A}$ and $\gamma_{e,B}$ denote the phenomenological decay rates of the electronically excited states of monomers A and B, respectively, capturing their finite lifetimes. In addition, $\gamma_{v,A}$ and $\gamma_{v,B}$ denote the decay rates of the vibrationally excited levels on the ground‐state potential energy surfaces of monomers A and B, respectively. Importantly, this framework can be straightforwardly extended to an arbitrary number of vibronic states on both the ground‐ and excited‐state potential energy surfaces (PES). Consequently, one can compute the linear absorption spectrum of any heterodimer using this protocol, without any constraint on the value of J, that is, the coupling need not be treated perturbatively. The only requirement is to parameterize the molecular dimer such that all parameters entering the Hamiltonian in Equation (2) are determined: specifically, the energies and wavefunctions of the vibronic states, and the dipole–dipole coupling strength J appearing in Equation (4). Although the linear response expressions in Equation (9) are exact, it is illuminating to explore certain perturbative regimes of this system, where J serves as the order parameter, as we do below.

We consider the linear response of a photosynthetically relevant heterodimer composed of Chl522 and Chl520, based on parameters summarized in Supporting Information S1: Table 1, obtained from ref. [50]. The corresponding absorption spectrum is given in Figure 1. Here, the absorption peak of Chl522 (A) monomer has an associated Raman (of Chl520 (B) monomer) sideband that is shifted by approximately the vibrational gap $\omega_{\nu ,B}=\omega_{g,1,B}-\omega_{g,0,B}=0.043\;\text{eV}$, and similarly, the absorption peak of Chl520 (B) monomer has an associated Raman (of Chl522 (A) monomer) sideband shifted by approximately $\omega_{\nu ,A}=\omega_{g,1,A}-\omega_{g,0,A}=0.043\;\text{eV}$. Thus, in this parameter regime, Raman‐type information is directly encoded in the linear absorption spectrum of the heterodimer. This interpretation is consistent with the intuition developed from the ladder diagrams presented in Supporting Information S1: Section 1: nonlinear contributions, specifically Raman‐type processes associated with the third‐order susceptibility, $\chi^{\left(3\right)}\left(\omega \right)$, are effectively encoded within the linear absorption spectrum of the dimer. This picture is further substantiated by the perturbative analysis presented in Supporting Information S1: Section 2, where the intermolecular coupling J in Equation (9) serves as the perturbative expansion parameter.

**FIGURE 1:**
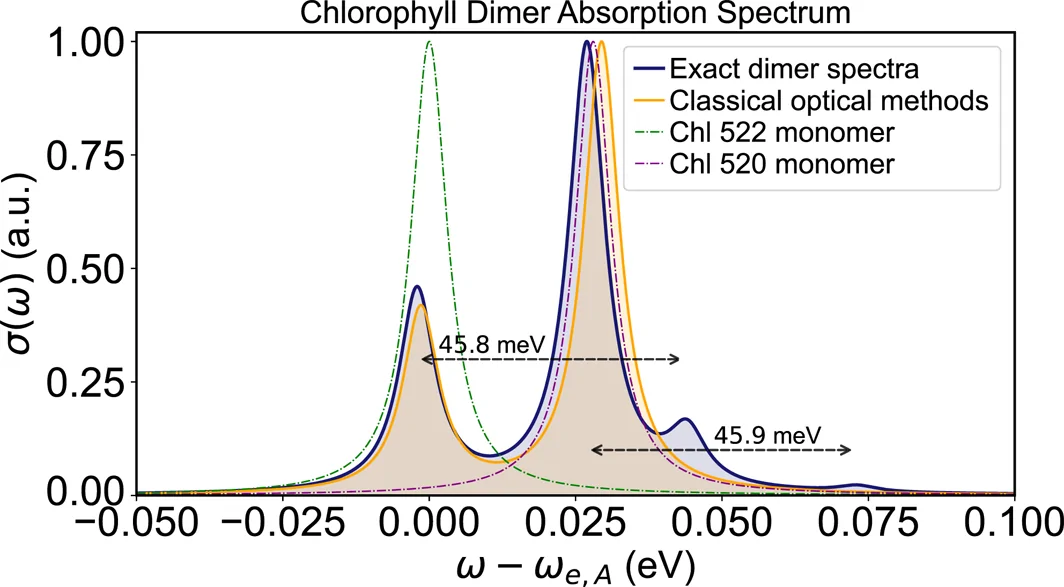
Absorption spectrum of the Chl522–Chl520 heterodimer computed using parameters from ref. [50] within a minimal three‐level system basis that captures the essential physics of the phenomenon. Details of the simulations and explicit parameters are provided in Supporting Information S1: Section 2. The blue curve shows the exact dimer spectrum obtained from Equation (9), whereas the orange curve corresponds to classical optical treatments (e.g., DDA/CPA/CES), which neglect contributions from ground‐state vibrational excitations to the linear response by restricting each monomer to a single ground vibrational level. The red dash–dot curve shows the normalized monomer absorption of Chl522, and the black dash–dot curve shows that of Chl520. Notably, the exact spectrum (blue) exhibits additional sidebands that are absent in the classical mean‐field descriptions. These features can be identified as Raman‐type sidebands: for instance, the third peak at $\omega -\omega_{e,A}\approx 0.05\;\text{eV}$ is shifted from the Chl522 monomer peak by approximately the vibrational frequency of Chl520, while the fourth peak at $\omega -\omega_{e,A}\approx 0.075\;\text{eV}$ is similarly shifted from the Chl520 monomer peak by the vibrational frequency of Chl522. The theoretical derivations and perturbative analysis supporting these assignments are presented in Supporting Information S1: Sections 1 and 2.

Here, the inter‐emitter interaction mediates exciton hopping that leaves behind phonons in the electronic ground state, thereby giving rise to this Raman‐type nonlinearity in the linear response of quantum‐emitter arrays. Thus, both the inter‐emitter interaction and their ability to generate ground‐state phonons within individual emitters are essential ingredients for observing this quantum‐optical phenomenon. Furthermore, the essential physics of this Raman‐type nonlinearity remains unchanged when additional vibrational levels are included in the model. Specifically, each additional ground‐state vibrational level gives rise to further Raman‐type sidebands around the classical optical peaks in Figure 1. In contrast, each additional excited‐state vibrational level produces additional classical optical peaks (vibronic progression) each of which have its own Raman side bands [3].

## 3 Linear Response in One‐Dimensional Quantum Emitter Arrays

In addition to the corrections observed in dimer spectra, these Raman processes also manifest as sidebands in the spectra of a strongly coupled quantum emitter array made up of 10 emitters, as shown in Figure 2. Here, we model the linear aggregate response using the standard shifted harmonic‐oscillator framework [3, 55]. These features are consistent with those reported by Roden et al. [56], who demonstrated, by varying the number of ground‐state vibrational levels, which spectral features are captured by the CES approximation and which lie beyond its scope.

**FIGURE 2:**
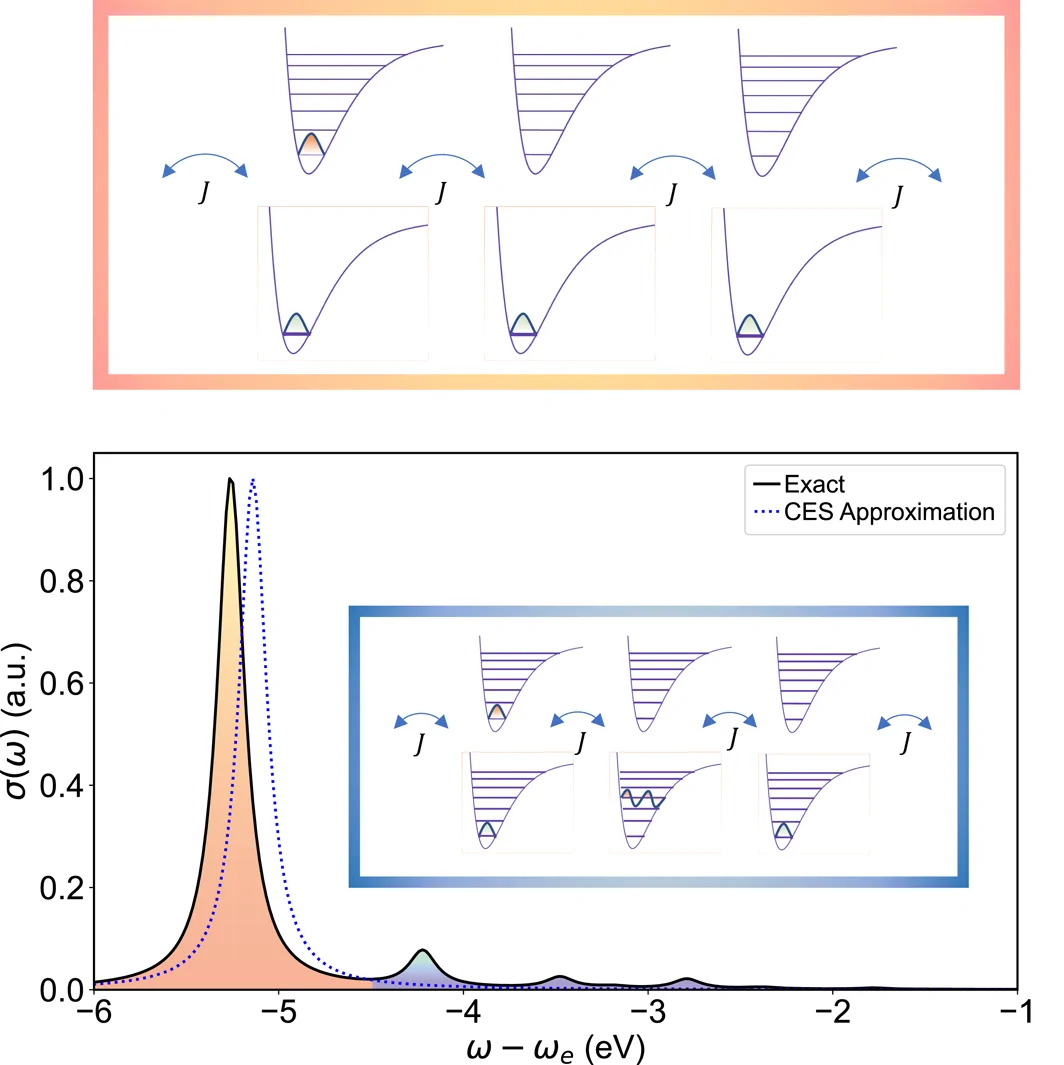
Linear absorption spectra of linearly coupled quantum emitter arrays comprising 10 emitters computed by exact numerical diagonalization. The details of the model and the parameters are presented in Supporting Information S1: Section 3. The DDA/CES/CPA captures the orange features in the exact spectra of the emitter arrays, analogous to those in Figure 1, which physically arises from Rayleigh‐type transitions, as schematically illustrated in the orange box. In contrast, spectral features such as the first blue‐shifted peak, displaced from the orange peak by precisely one quantum of the ground‐state vibrational frequency, are entirely absent within the DDA/CES/CPA approximations, in agreement with Figure 1 and originating from Raman‐type transitions, as depicted in the blue schematic. Moreover, the spectra exhibit additional blue‐shifted combination bands, approximately spaced by integer multiples of the vibrational frequency. We attribute this progression to higher‐order photophysical pathways involving multiple monomers that host vibrational excitations in their ground electronic manifolds. Collectively, these features expose hidden nonlinear optical and vibronic correlations beyond the scope of CES and underscore the role of Raman‐active processes in shaping the aggregate response. The simulation parameters are provided in Supporting Information S1: Section 3.

In this emitter array setup, our analysis of the dimer spectra provides a clear physical interpretation of these previously unexplained sidebands. We show that they originate from Raman‐active vibrational modes of the monomer, which emerge in the linear spectra of the emitter arrays as sidebands. Additionally, the spectra display blue‐shifted combination bands, spaced approximately by integer multiples of the vibrational frequency. We attribute this progression to higher‐order photophysical pathways involving multiple monomers carrying vibrational excitations in their ground electronic states. Collectively, these insights reveal that linear absorption spectra of quantum emitter arrays encode richer vibrational information than previously appreciated, thereby deepening the structure–spectra relationship. Consequently, aggregate spectra can be exploited not only to probe excitonic couplings but also to infer ground‐state vibrational fingerprints of the constituent monomers. Similarly, our results suggest a pathway for experimentally tuning aggregate spectral features through controlled modification of ground‐state vibrational structure. This result is also consistent with the many‐particle approximations [57] used by Spano et al. [58, 59] to model aggregate absorption spectra. In particular, in certain strong‐coupling regimes, these theories predict the emergence of side peaks (Figure 17j in ref: [3]), which we interpret as Raman signatures (Supporting Information S1: Section 4).

Note that the dominant spectral peaks in both Figures 1 and 2 are red‐shifted relative to their classical counterparts. This shift arises because the classical optical treatment neglects the Raman‐type mechanism identified here. For example, it omits couplings between $|e_{A},0_{A}^{\prime };g_{B},0_{B}\rangle$ and $|g_{A},1_{A};e_{B},0_{B}^{\prime }\rangle$. In contrast, these couplings are retained in our exact spectra, where the resulting level repulsion produces a red shift relative to the uncoupled classical optical peaks.

Further, the parameters used in Figure 2 are similar to those previously explored in the literature [3, 56] to examine spectra across different theoretical coupling regimes (rather than a specific experimentally characterized system, unlike the heterodimer spectrum in Figure 1).

In the present analysis, we have not explicitly incorporated inhomogeneous broadening. Nevertheless, our conclusions are expected to remain representative of experimentally relevant spectra [3, 56, 58] in the regime $W>\omega_{v}>\sigma$, where W is the exciton bandwidth, $\omega_{v}$ is the Raman vibrational frequency, and σ is decay rate associated with the inhomogeneous broadening. This hierarchy defines an especially favorable window for observing Raman sidebands: the emitter array coupling is sufficiently strong compared to vibronic interactions to generate well‐defined collective resonances, yet not so strong that the system reduces to the simple excitonic picture captured by Kasha's theory (vibronic decoupling [3]) [60, 61]. Moreover, the dominant aggregate absorption peaks, being significantly shifted from their monomer counterparts are known to exhibit reduced sensitivity to inhomogeneous broadening [62, 63]. Because the Raman features identified here appear in the vicinity of these robust aggregate resonances, we anticipate that disorder‐induced broadening plays only a minor role in obscuring these spectral signatures within the regime of interest.

## 4 Conclusion

In this work, by analyzing the linear response of coupled heterodimers and extending the framework to arrays of quantum emitters, we uncover the emergence of hidden optical nonlinearities encoded within the linear spectra of coupled systems.

Related features were recently identified in the distinct setting of cavity–matter coupled system [40], but important differences remain. First, in such systems, the features are mediated by microresonators, whereas in quantum‐emitter arrays they are mediated by intermolecular coupling. Second, their intensity in the cavity setup scales with orders of single‐molecule light–matter coupling, g, which is typically smaller than the cavity and molecular decay rates. High‐Q resonators are therefore required to resolve these rare events. In our system, the strength instead scales with the intermolecular coupling J, which is often much larger than g. Consequently, the features in Figure 1 can be comparable in intensity to the classical optical peaks, unlike those in the cavity setting. Finally, Figures 1 and 2 show that permutational symmetry is not required, unlike in the previously reported cavity‐mediated case. This implies that these features are not subject to the 1/N suppression characteristic of symmetric systems [64, 65], allowing them to persist at appreciable strength even in large ensembles. Thus, Raman‐type information traditionally obtained through nonlinear spectroscopy is embedded here in the linear absorption spectra of coupled emitters, which are routinely measured and well established in molecular‐aggregate literature.

Importantly, we identify experimentally relevant parameter regimes, particularly within the chlorophyll heterodimer and molecular aggregate literature that have been largely overlooked, yet exhibit clear Raman sideband signatures adjacent to the primary absorption peaks. Our analysis demonstrates that linear absorption spectra of molecular aggregates encode detailed ground‐state vibrational information, thereby providing a principled route toward spectral engineering via controlled modification of monomer vibrational structure. Notably, these Raman‐derived features are also captured by established many‐body approaches, such as the two‐particle approximation (TPA) [58], reinforcing their physical robustness. Finally, we anticipate that these signatures will remain experimentally observable in regimes of strong inter‐emitter coupling, where disorder‐resilient collective resonances preserve adjacent Raman sidebands.

At a broader level, Raman spectroscopy has been established as a cornerstone of molecular characterization because it interrogates higher‐order molecular susceptibilities that are fundamentally inaccessible to linear optical measurements. In this work, we demonstrate that such higher‐order information of the monomers can naturally emerge as sidebands in the linear spectra of quantum emitter arrays. Remarkably, the underlying inter‐emitter coupling, mediated by the electromagnetic vacuum, enables effective higher‐order processes involving Raman‐active vibrational modes, even in the presence of weak optical driving that cannot directly access these modes. This mechanism reveals a new pathway for exploiting inter‐emitter interactions to probe individual emitter degrees of freedom that remain hidden to a weak laser field interacting with uncoupled emitters. Crucially, this physics lies beyond the scope of conventional classical‐optics‐based DDA/CES/CPA approaches, which model the linear spectra of coupled systems solely through the linear susceptibilities of their individual constituents.

Moreover, we utilized a novel Dyson resummation approach to compute the linear response of quantum emitter arrays. This approach offers a particularly clean route to describing the linear response of the coupled system compared with diagonalizing the full vibronic Hamiltonian. Each order in the expansion admits a transparent photophysical interpretation, whereas resummation of the full series would yield the same spectra as exact diagonalization. This structure makes the approach especially attractive for many‐pigment systems, where the cost of explicit diagonalization becomes prohibitive.

Looking ahead, our work identifies specific regimes in quantum emitter array systems where informative Raman signatures emerge directly from the array linear optical response, thereby enabling experimental access to and characterization of the underlying monomeric units. Beyond spectroscopy, these results introduce a new and previously unexplored avenue for tunability in the design of efficient optoelectronic devices, namely through ground‐state Raman‐active vibrational modes that can directly influence the photophysical properties of the emitter array. More broadly, our results contribute to the growing body of genuinely quantum‐mechanical phenomena in molecular aggregates by explicitly accounting for vibronic degrees of freedom. In the absence of such couplings, it has been argued that classical and quantum‐coherent descriptions of emitter array dynamics become effectively indistinguishable [54]. In contrast, we show that the inclusion of molecular vibrations gives rise to distinctly quantum signatures that persist even in the linear response regime, traditionally regarded as adequately described by classical multichromophoric frameworks [52]. Because these effects originate from nonclassical inter‐monomer correlations generated by inter‐emitter coupling, we interpret the resulting Raman‐induced spectral features as signatures of monomer–monomer entanglement, a connection that we plan to investigate quantitatively in future work. Moreover, our result belongs to the tradition of molecular aggregate spectroscopy, in which microscopic insights are used to infer and ultimately engineer aggregate geometry to tune photophysical properties. Identifying concrete optoelectronic manifestations of this genuinely quantum‐optical effect will be the subject of future work. Altogether, our results open new directions in the study of quantum emitter arrays by demonstrating how inter‐emitter interactions can harvest otherwise hidden optical nonlinearities, giving rise to emergent quantum effects and providing a new control knob for optimizing optoelectronic and quantum sensing performance.

## Author Contributions

**Sricharan Raghavan‐Chitra:** conceptualization, methodology, formal analysis, investigation, visualization, writing – review and editing, writing – original draft. **Arghadip Koner:** conceptualization, writing – review and editing, methodology. **Joel Yuen‐Zhou:** conceptualization, funding acquisition, writing – review and editing, writing – original draft, supervision, project administration.

## Conflicts of Interest

The authors declare no conflicts of interest.

## Supporting information


Supporting Information S1

## Data Availability

Data sharing is not applicable to this article as no new data were created or analyzed in this study.
