# Supplementary material for: Hidden Optical Nonlinearities in Linear Spectra of Quantum Emitter Arrays
Source: Nanophotonics. 2026 Aug 19;15(16):e70252. doi: 10.1002/nap2.70252 (PMC13672167; doi:10.1002/nap2.70252)
Supplement: Supplementary file 1 [file NAP2-15-e70252-s001.pdf]

Supplementary Information: Hidden optical nonlinearities in linear spectra of quantum emitter arrays

Sricharan Raghavan-Chitra, Arghadip Koner, Joel Yuen-Zhou*

August 15, 2026

Department of Chemistry and Biochemistry, University of California San Diego, La Jolla, California 92093

Contents

1 Diagonal and off-diagonal terms in Eq. 9 of the main text **1**

1.1 Diagonal terms . . . 1

1.1.1 Zeroth-order term: $\langle e_A, 0'_A; g_B, 0_B|\hat{G}_0(\omega)|e_A, 0'_A; g_B, 0_B\rangle$ . . . 2

1.1.2 First-order term: $\langle e_A, 0'_A; g_B, 0_B|\hat{G}_0\hat{V}\hat{G}_0|e_A, 0'_A; g_B, 0_B\rangle$ . . . 2

1.1.3 Second-order term: $\langle e_A, 0'_A; g_B, 0_B|\hat{G}_0\hat{V}\hat{G}_0\hat{V}\hat{G}_0|e_A, 0'_A; g_B, 0_B\rangle$ . . . 3

1.1.4 Fourth-order term: $\langle e_A, 0'_A;\, g_B, 0_B\,|\,\hat{G}_0(\omega)\,\hat{V}\,\hat{G}_0(\omega)\,\hat{V}\,\hat{G}_0(\omega)\,\hat{V}\,\hat{G}_0(\omega)\,\hat{V}\,\hat{G}_0(\omega)\,|\,e_A, 0'_A;\, g_B, 0_B\rangle$ . . . 3

1.1.5 Resummation . . . 5

1.2 Off diagonal terms in Eq. 9 of the main text . . . 6

1.2.1 Zeroth order: $\langle e_A, 0'_A; g_B, 0_B|G_0\,|g_A, 0_A; e_B, 0'_B\rangle$ . . . 6

1.2.2 First order: $\langle e_A, 0'_A; g_B, 0_B|G_0\hat{V}G_0\,|g_A, 0_A; e_B, 0'_B\rangle$ . . . 6

1.2.3 Resummation . . . 6

1.3 Final expression for the absorption spectrum of the dimer . . . 7

2 Heterodimer simulation **8**

3 Linear response of linear molecular aggregates **10**

4 Many particle approximations **11**

1 Diagonal and off-diagonal terms in Eq. 9 of the main text

In this section, we systematically evaluate both the diagonal and off-diagonal matrix elements appearing on the right-hand side of Eq. (9) of the main text. The section is organized as follows: Sec. 1.1 is devoted to the computation of the diagonal contributions, while Sec. 1.2 addresses the corresponding off-diagonal contributions.

1.1 Diagonal terms

In this subsection, we compute the diagonal terms in Eq. 9 of the main text,

$$\langle e_A, 0'_A;\, g_B, 0_B\,|\,\hat{G}(\omega)\,|\,e_A, 0'_A;\, g_B, 0_B\rangle, \tag{S1}$$

via the Dyson expansion, by partitioning the full Hamiltonian as $\hat{H} = \hat{H}_0 + \hat{V}$, where $\hat{H}_0 = \hat{H}_A + \hat{H}_B$ denotes the bare, non-interacting Hamiltonian, and $\hat{V} = \hat{H}_I$ is the excitonic coupling, treated as a perturbation. The Dyson expansion [3] then yields:

$$\begin{aligned}\langle e_A, 0'_A;\, g_B, 0_B\,|\,\hat{G}(\omega)\,|\,e_A, 0'_A;\, g_B, 0_B\rangle &= \langle e_A, 0'_A;\, g_B, 0_B\,|\,\hat{G}_0(\omega)\,|\,e_A, 0'_A;\, g_B, 0_B\rangle \\ &+ \langle e_A, 0'_A;\, g_B, 0_B\,|\,\hat{G}_0(\omega)\,\hat{V}\,\hat{G}_0(\omega)\,|\,e_A, 0'_A;\, g_B, 0_B\rangle \\ &+ \langle e_A, 0'_A;\, g_B, 0_B\,|\,\hat{G}_0(\omega)\,\hat{V}\,\hat{G}_0(\omega)\,\hat{V}\,\hat{G}_0(\omega)\,|\,e_A, 0'_A;\, g_B, 0_B\rangle + \cdots,\end{aligned} \tag{S2}$$

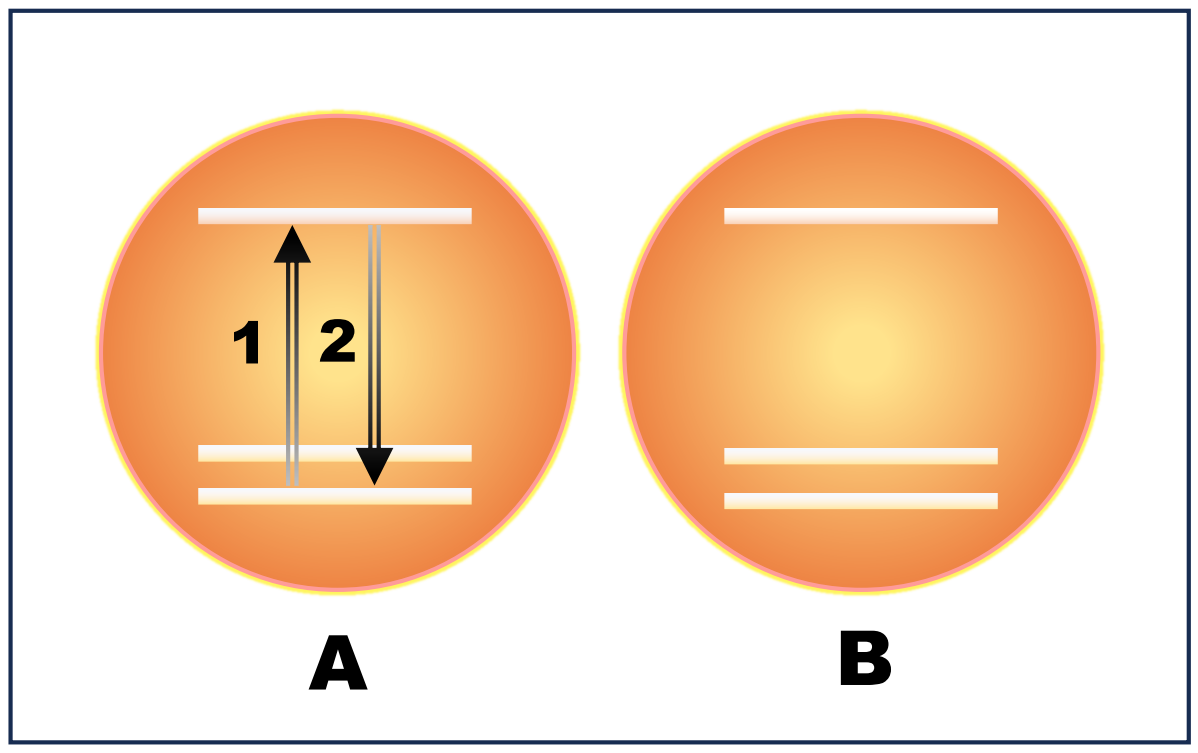

Figure S1: The ladder diagram [8] of the heterodimer illustrates the photophysical process in which the incident light field interacts perturbatively with monomer A alone. The double-line arrows denote processes directly mediated by the incoming laser. The numbers adjacent to each arrow indicate the sequence of interactions: (1) excitation of monomer A by the laser and (2) de-excitation of monomer A through a second interaction with the laser.

where $\hat{G}_0(\omega)$ is the retarded Green's function of the non-interacting system:

$$\hat{G}_0(\omega) = \frac{1}{\omega - \hat{H}_0 + i0^+}. \tag{S3}$$

The successive terms on the right-hand side of Eq. S2 are referred to as the zeroth-order, first-order, second-order contributions, and so on, in the standard sense of the Dyson expansion in powers of $\hat{V}$. We emphasize that this notion of "order" is distinct from its usage in previous works from our research group based on the CUT-E method ([5, 4]). In the following subsections, we systematically compute each of these terms order by order.

1.1.1 Zeroth-order term: $\langle e_A, 0'_A; g_B, 0_B|\hat{G}_0(\omega)|e_A, 0'_A; g_B, 0_B\rangle$

The zeroth-order contribution is straightforwardly evaluated as:

$$\langle e_A, 0'_A; g_B, 0_B|\hat{G}_0(\omega)|e_A, 0'_A; g_B, 0_B\rangle = \frac{1}{\omega - (\omega_{e,0,A} + \omega_{g,0,B}) + i\frac{\gamma_{e,A}}{2}}, \tag{S4}$$

where $\gamma_{e,A}$ is the decay rate associated with the electronic excited state of monomer A, introduced phenomenologically to account for the finite lifetime of the excited state. For notational compactness, we define:

$$G_{e,A} \equiv \frac{1}{\omega - (\omega_{e,0,A} + \omega_{g,0,B}) + i\frac{\gamma_{e,A}}{2}}. \tag{S5}$$

Physically, this zeroth-order term describes the free propagation of the excitation on monomer A, in the absence of any inter-monomer coupling. The corresponding photophysical process is depicted in the ladder diagram in Fig. S1, where the double-line arrow represents processes directly mediated by the incoming laser, with no contribution from the perturbation $\hat{V}$.

1.1.2 First-order term: $\langle e_A, 0'_A; g_B, 0_B|\hat{G}_0\hat{V}\hat{G}_0|e_A, 0'_A; g_B, 0_B\rangle$

The first-order contribution to the Dyson expansion evaluates as:

$$\langle e_A, 0'_A; g_B, 0_B|\hat{G}_0\hat{V}\hat{G}_0|e_A, 0'_A; g_B, 0_B\rangle = G_{e,A}^2 \langle e_A, 0'_A; g_B, 0_B|\hat{V}|e_A, 0'_A; g_B, 0_B\rangle = 0. \tag{S6}$$

This vanishes because the interaction Hamiltonian $\hat{V} = H_I$ couples states of the form $|e_A; g_B\rangle \leftrightarrow |g_A; e_B\rangle$, and therefore has no diagonal matrix elements in this basis. By the same argument, all odd-order terms in the Dyson expansion of Eq. (S2) vanish identically, since each such term necessarily contains an odd power of $\hat{V}$ sandwiched between the same bra and ket, which cannot be connected by an odd number of excitation-transfer operations.

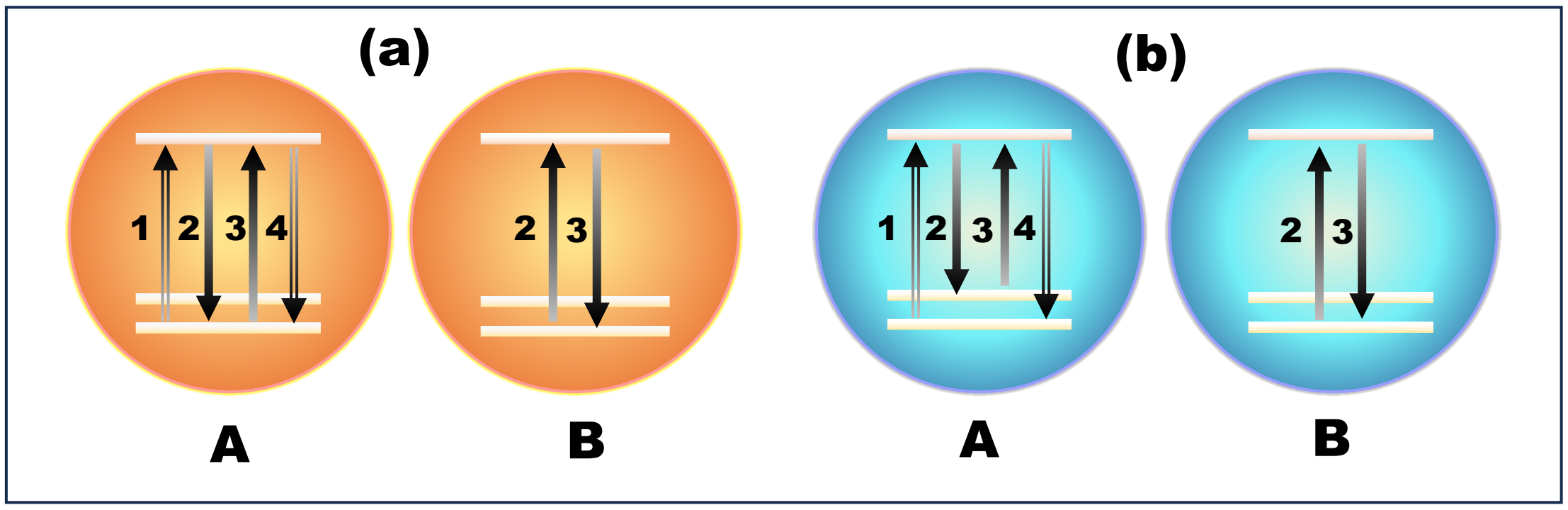

Figure S2: The ladder diagram [8] corresponding to the second-order term in Eq. S2 illustrates the case where the light field interacts perturbatively with monomer A alone. Both diagrams involve two successive dipole-dipole interactions, through which the excitation is transferred to monomer B and subsequently returned to monomer A. Panel (a) captures a purely Rayleigh-type photophysical process, with no vibrational excitation in the ground electronic state, whereas panel (b) includes a Raman-type contribution in which vibrational coherence is generated in monomer A. The double-line arrows denote processes directly mediated by the incoming laser, while the solid arrows represent dipole-dipole (J-coupling) mediated processes. The numbers adjacent to each arrow indicate the sequence of interactions: (1) excitation of monomer A by the laser, (2–3) exciton transfer from A to B via dipole-dipole coupling, and (4) de-excitation of monomer A through a second interaction with the laser.

1.1.3 Second-order term: $\langle e_A, 0'_A; g_B, 0_B|\hat{G}_0\hat{V}\hat{G}_0\hat{V}\hat{G}_0|e_A, 0'_A; g_B, 0_B\rangle$

The leading non-vanishing contribution in the Dyson expansion that involves the coupling $\hat{V}$ arises at second order. Evaluating explicitly, we find:

$$\langle e_A, 0'_A;\, g_B, 0_B \,|\, \hat{G}_0(\omega)\, \hat{V}\, \hat{G}_0(\omega)\, \hat{V}\, \hat{G}_0(\omega) \,|\, e_A, 0'_A;\, g_B, 0_B\rangle = J^2\, G^2_{e,A}\, (G_{e,B} + G_{v,A,e,B})\,, \tag{S7}$$

where we have defined:

$$G_{e,B} \equiv \frac{1}{\omega - (\omega_{e,0,B} + \omega_{g,0,A}) + i\,\dfrac{\gamma_{e,B}}{2}}, \tag{S8}$$

$$G_{v,A,e,B} \equiv \frac{1}{\omega - (\omega_{e,0,B} + \omega_{g,1,A}) + i\,\dfrac{\gamma_{e,B} + \gamma_{v,A}}{2}}. \tag{S9}$$

Here, $\gamma_{e,B}$ is the decay rate of the electronic excited state of monomer B, and $\gamma_{v,A}$ is the decay rate of the vibrationally excited level on the ground-state potential energy surface (PES) of monomer A.

The physical content of this result is particularly illuminating. The factor J^2 reflects the second-order nature of the perturbation $\hat{V}$. The factor $G^2_{e,A}$ accounts for two free propagations of the system in the state $|e_A, 0'_A;\, g_B, 0_B\rangle$ — once immediately following the first laser interaction, and once before the second. Notably, since this term is second order in $\hat{V}$, monomer A exchanges the exciton twice with monomer B, returning to A before the second laser interaction takes place. During this exciton exchange, two distinct pathways are available. First, the exciton is exchanged with B without depositing any phonon in the ground state of A — this is referred to as the **Rayleigh process** (depicted in Fig. S2(a)). Second, the exciton is exchanged with B while leaving a phonon in the ground state of A — this is referred to as the **Raman process** (depicted in Fig. S2(b)). These two pathways are captured in the factor $(G_{e,B} + G_{v,A,e,B})$.

Remarkably, we already observe that a process known to capture the nonlinear susceptibility of the monomer is contributing to the *linear* response of the dimer. This emerges as a recurring theme throughout the derivation: at each successive even order of perturbation theory, additional Raman-type processes appear, progressively encoding the nonlinear response of the individual monomers into the linear response of the dimer.

1.1.4 Fourth-order term: $\langle e_A, 0'_A;\, g_B, 0_B \,|\, \hat{G}_0(\omega)\, \hat{V}\, \hat{G}_0(\omega)\, \hat{V}\, \hat{G}_0(\omega)\, \hat{V}\, \hat{G}_0(\omega)\, \hat{V}\, \hat{G}_0(\omega) \,|\, e_A, 0'_A;\, g_B, 0_B\rangle$

The diagrams shown in Fig. S2 serve two purposes. First, they make explicit the underlying photophysical processes induced by the laser interactions, represented by double-line arrows, and by the dipole–dipole interactions, represented by solid arrows. This representation is particularly useful for identifying pathways that encode Raman-type information. Second, the diagrams

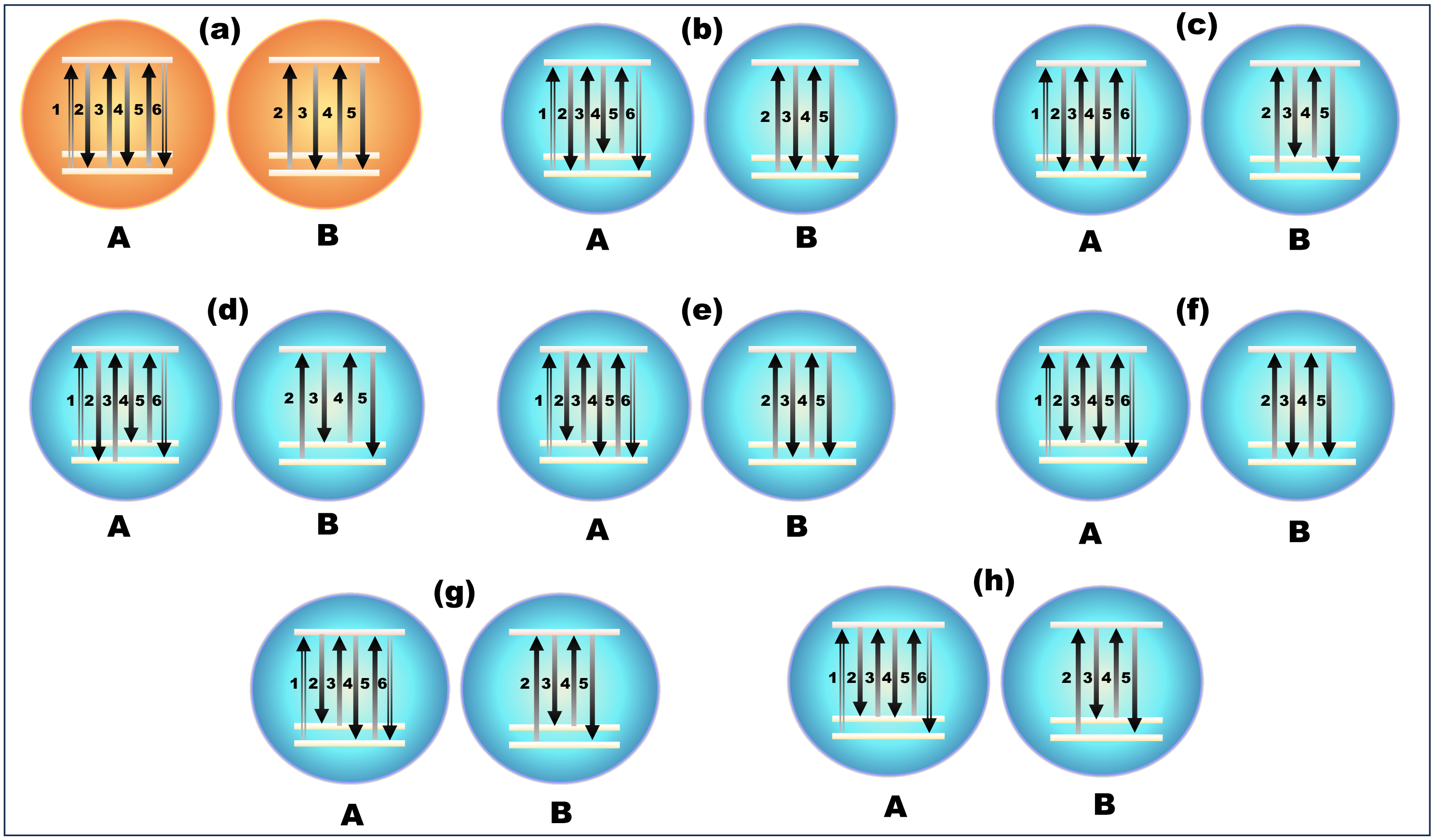

Figure S3: All possible ladder diagrams [8] are shown corresponding to fourth order in the interaction between the monomers. Further, these diagrams correspond to the component where the light source interacts only with monomer A. Only Rayleigh-type photophysical processes takes place in the orange diagram (a), whereas, Raman-type processes also takes place in the blue diagrams (b-h). Here, the double line arrows correspond to direct processes due to the laser and the solid line arrows correspond to processes due to the dipole-dipole interaction (J- coupling). The numbers adjacent to each arrow indicate the sequence of interactions: (1) excitation of monomer A by the laser, (2–5) exciton transfer from A to B via dipole-dipole coupling, and (6) de-excitation of monomer A through a second interaction with the laser.

provide a systematic bookkeeping device for generating all possible processes at a given order in perturbation theory. There is a one-to-one correspondence between these ladder diagrams and Double-Sided Feynman Diagrams, as explored in ref: [2], but we believe it might be simpler for the reader to understand the former in case they are not familiar with the latter.

In particular, during exciton exchange between the two monomers, two possibilities always arise: either the monomer that loses the exciton is left without a vibrational excitation, or it is left with a vibrational excitation. Consequently, the diagrammatic construction offers a transparent way of enumerating all allowed contributions at a fixed perturbative order.

This procedure is illustrated for the fourth-order contribution in Eq. S2,

$$\langle e_A, 0'_A;\, g_B, 0_B \,|\, \hat{G}_0(\omega)\, \hat{V}\, \hat{G}_0(\omega)\, \hat{V}\, \hat{G}_0(\omega)\, \hat{V}\, \hat{G}_0(\omega)\, \hat{V}\, \hat{G}_0(\omega) \,|\, e_A, 0'_A;\, g_B, 0_B \rangle \tag{S10}$$

in Fig. S3. In the present minimal model, the role of the interaction operator $\hat{V}$ is solely to exchange the exciton between the two monomers, while contributing a factor of J at each application. All energy denominators, and hence all poles of the response, originate from the free propagator $\hat{G}_0(\omega)$. Thus, once a diagram is specified, its analytic expression can be straightforwardly obtained by reading off the sequence of intermediate states generated after each interaction and writing down the corresponding free propagators in the same order.

The numbers attached to the arrows in Fig. S3 indicate the sequence of interactions, to be read from left to right in Eq. S10. It is important to note that the bra and ket appearing in Eq. S10 already correspond to the state produced after the initial laser interaction, which is depicted by the double-line arrows in Fig. S3. Accordingly, Fig. S3 contains all possible diagrams generated by the repeated action of $\hat{V}$, with the initial and final laser interactions held fixed. In the present case, both of these laser interactions occur on monomer A.

To illustrate the protocol, consider a specific diagram, Fig. S3(b). Its analytic contribution is obtained by recording the free-propagation factor associated with each intermediate state after every interaction, including the first laser-induced excitation. For the diagram under consideration, this yields

$$G_{e,A}\,J\,G_{e,B}\,J\,G_{e,A}\,J\,G_{e,B,v,A}\,J\,G_{e,A}, \tag{S11}$$

where the ordering of the propagators follows directly the ordering of the arrows in the diagram.

Repeating this procedure for all fourth-order diagrams and summing the resulting contributions gives

$$\begin{aligned}&\langle e_A, 0'_A;\, g_B, 0_B\,|\,\hat{G}_0(\omega)\,\hat{V}\,\hat{G}_0(\omega)\,\hat{V}\,\hat{G}_0(\omega)\,\hat{V}\,\hat{G}_0(\omega)\,\hat{V}\,\hat{G}_0(\omega)\,|\,e_A, 0'_A;\, g_B, 0_B\rangle \\ &= J^2 G_{e,A}^2 (G_{e,B} + G_{v,A,e,B})\, J^2 (G_{e,A} + G_{e,A,v,B})(G_{e,B} + G_{v,A,e,B}).\end{aligned} \tag{S12}$$

One can rationalize this structure as follows. The prefactor $J^2 G_{e,A}^2\,(G_{e,B} + G_{v,A,e,B})$ has already been established in the analysis of the second-order contribution (Sec. 1.1.3), where it arises due to the constraint that the laser interacts exclusively with monomer A. In contrast, the two additional applications of the interaction operator $\hat{V}$, corresponding to arrows 3 and 4 in Fig. S3(b), are unconstrained and free to generate every intermediate state compatible with exciton exchange between the two monomers within the present minimal model. As a result, the remaining contributions organize into the factor $J^2\,(G_{e,A} + G_{e,A,v,B})\,(G_{e,B} + G_{v,A,e,B})$, where the prefactor J^2 arises from the two additional applications of $\hat{V}$. The term $(G_{e,A} + G_{e,A,v,B})$ enumerates all possible configurations following an exciton transfer from monomer B to monomer A, while $(G_{e,B} + G_{v,A,e,B})$ collects the corresponding configurations following an exciton transfer from monomer A to monomer B.

1.1.5 Resummation

One can now recognize the emerging structure of the perturbation series. In particular, by following the same diagrammatic logic used at fourth order, one finds that the sixth-order contribution takes the form

$$J^2 G_{e,A}^2 (G_{e,B} + G_{v,A,e,B}) \big[J^2 (G_{e,A} + G_{e,A,v,B})(G_{e,B} + G_{v,A,e,B})\big]^2.$$

The key point is that each additional pair of interactions with $\hat{V}$ contributes the same building block,

$$J^2 (G_{e,A} + G_{e,A,v,B})(G_{e,B} + G_{v,A,e,B}),$$

which accounts for all allowed intermediate excitonic and vibronic configurations generated by one further round-trip of the exciton between monomers A and B. Thus, the perturbation series assumes a geometric structure, allowing all orders in $\hat{V}$ to be resummed exactly.

As a result, the exact response corresponding to $\langle e_A, 0'_A;\, g_B, 0_B\,|\,\hat{G}(\omega)\,|\,e_A, 0'_A;\, g_B, 0_B\rangle$ (in Eq. 9 of the main text) can be written as

$$\langle e_A, 0'_A;\, g_B, 0_B\,|\,\hat{G}(\omega)\,|\,e_A, 0'_A;\, g_B, 0_B\rangle = G_{e,A} + J^2 G_{e,A}^2 (G_{e,B} + G_{v,A,e,B}) \sum_{n=0}^{\infty} \Big[J^2 (G_{e,A} + G_{e,A,v,B})(G_{e,B} + G_{v,A,e,B})\Big]^n.$$

Since this is a geometric series, it may be resummed immediately to give

$$\langle e_A, 0'_A;\, g_B, 0_B\,|\,\hat{G}(\omega)\,|\,e_A, 0'_A;\, g_B, 0_B\rangle = G_{e,A} + \frac{J^2 G_{e,A}^2 (G_{e,B} + G_{v,A,e,B})}{1 - J^2 (G_{e,A} + G_{e,A,v,B})(G_{e,B} + G_{v,A,e,B})}.$$

Upon combining terms, this expression can be recast in the compact form

$$\langle e_A, 0'_A;\, g_B, 0_B\,|\,\hat{G}(\omega)\,|\,e_A, 0'_A;\, g_B, 0_B\rangle = \frac{G_{e,A}\Big[1 - J^2 G_{e,A,v,B}(G_{e,B} + G_{v,A,e,B})\Big]}{1 - J^2 (G_{e,A} + G_{e,A,v,B})(G_{e,B} + G_{v,A,e,B})}.$$

By symmetry under interchange of the monomer labels $A \leftrightarrow B$, one immediately obtains the corresponding diagonal matrix element for the configuration in which the exciton resides on monomer B (refer Eq. 9 of the main text), namely

$$\langle e_B, 0'_B;\, g_A, 0_A\,|\,\hat{G}(\omega)\,|\,e_B, 0'_B;\, g_A, 0_A\rangle = \frac{G_{e,B}\Big[1 - J^2 G_{v,A,e,B}(G_{e,A} + G_{e,A,v,B})\Big]}{1 - J^2 (G_{e,B} + G_{v,A,e,B})(G_{e,A} + G_{e,A,v,B})}.$$

1.2 Off diagonal terms in Eq. 9 of the main text

Following the procedure for the diagonal elements mentioned in Sec. 1.1, we can compute the exact expression for the off diagonal elements, namely, $\langle e_A, 0'_A; g_B, 0_B| \hat{G}(\omega) |g_A, 0_A; e_B, 0'_B\rangle$ using dyson expansion and ladder diagrams. Thus,

$$\begin{aligned}\langle e_A, 0'_A; g_B, 0_B|G(\omega) |g_A, 0_A; e_B, 0'_B\rangle = &\langle e_A, 0'_A; g_B, 0_B|G_0 |g_A, 0_A; e_B, 0'_B\rangle \\ &+ \langle e_A, 0'_A; g_B, 0_B|G_0\hat{V}G_0 |g_A, 0_A; e_B, 0'_B\rangle \\ &+ \langle e_A, 0'_A; g_B, 0_B|G_0\hat{V}G_0\hat{V}G_0 |g_A, 0_A; e_B, 0'_B\rangle \\ &+ \dots \end{aligned} \tag{S13}$$

1.2.1 Zeroth order: $\langle e_A, 0'_A; g_B, 0_B|G_0 |g_A, 0_A; e_B, 0'_B\rangle$

Here, the zeroth-order term is

$$\langle e_A, 0'_A; g_B, 0_B|G_0|g_A, 0_A; e_B, 0'_B\rangle = 0,$$

and, more generally, all even-order terms vanish. The reason is that each application of the perturbation exchanges the exciton between the two monomers. Therefore, after an even number of such interactions, the exciton necessarily returns to the monomer on which it was initially localized. As a result, even-order processes cannot contribute to off-diagonal matrix elements in this basis.

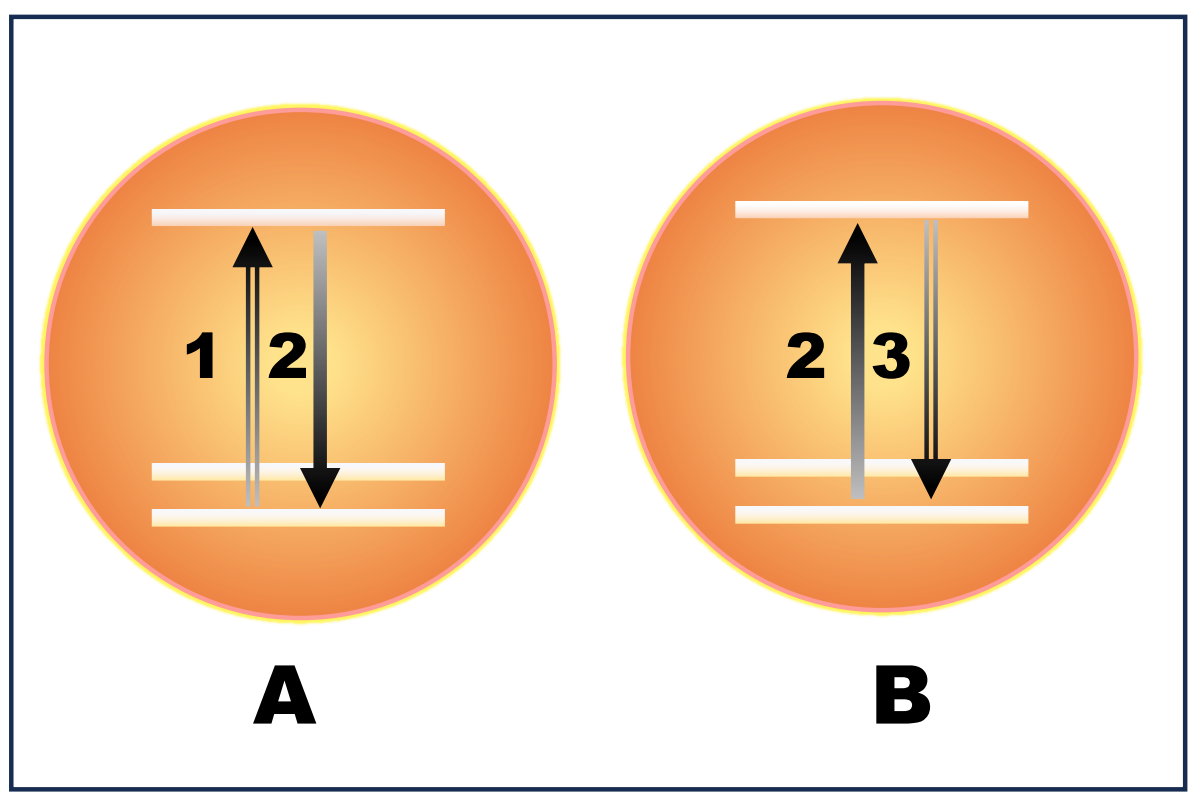

Figure S4: The ladder diagram [8] represents the pathway in which the light field excites monomer A and subsequently de-excites monomer B. This diagram corresponds to a first-order interaction process. The double-line arrows denote direct interactions with the laser field, while the solid arrows represent dipole-dipole (J-coupling) mediated processes. The numbers adjacent to each arrow indicate the sequence of interactions: (1) excitation of monomer A by the laser, (2) exciton transfer from A to B via dipole-dipole coupling, and (3) de-excitation of monomer B through a second interaction with the laser.

1.2.2 First order: $\langle e_A, 0'_A; g_B, 0_B|G_0\hat{V}G_0 |g_A, 0_A; e_B, 0'_B\rangle$

The first-order term is

$$\langle e_A, 0'_A; g_B, 0_B|G_0\hat{V}G_0|g_A, 0_A; e_B, 0'_B\rangle = JG_{e,A}G_{e,B}.$$

The corresponding photophysical process is illustrated by the ladder diagrams in Fig. S4. Here, the diagrammatic intuition developed in Sec. 1.1.4 is borne out transparently: the factor J arises from the single application of the perturbation $\hat{V}$, while $G_{e,A}$ and $G_{e,B}$ describe the free propagation of the system before and after the exciton is exchanged from monomer B to monomer A, respectively.

1.2.3 Resummation

The photophysical processes contributing to the third-order term are illustrated by the ladder diagrams in Fig. S5. Summing these diagrams yields

$$JG_{e,A}G_{e,B}\, J^2(G_{e,A} + G_{e,A,v,B})(G_{e,B} + G_{v,A,e,B}). \tag{S14}$$

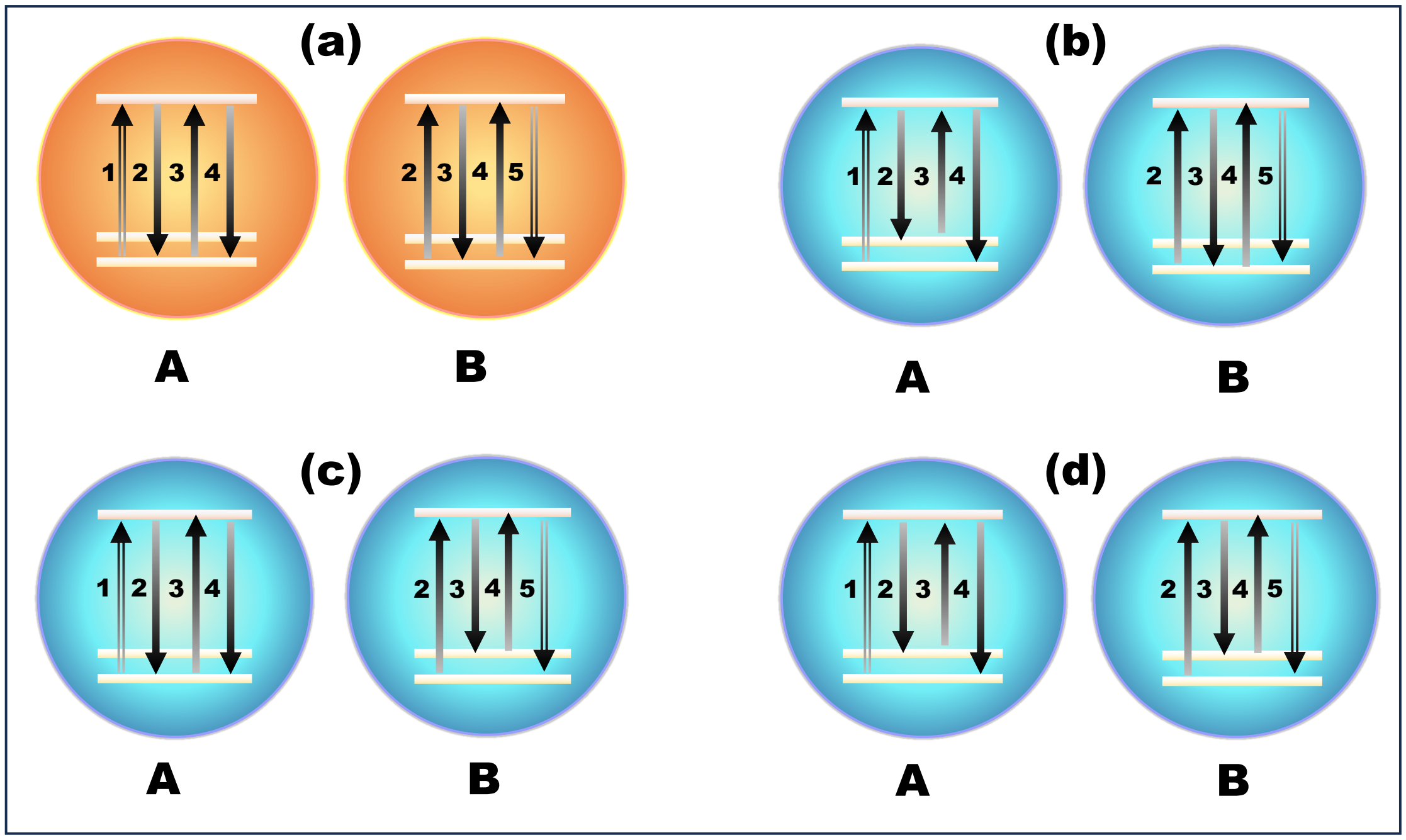

Figure S5: The ladder diagram [8] represents the pathway in which the light field excites monomer A and subsequently de-excites monomer B. This diagram corresponds to a third-order interaction process. The orange diagram (a) captures only Rayleigh-type processes, whereas the blue diagrams (b–d) additionally include Raman-type processes. The double-line arrows denote direct interactions with the laser field, while the solid arrows represent dipole-dipole (J-coupling) mediated processes. The numbers adjacent to each arrow indicate the sequence of interactions: (1) excitation of monomer A by the laser, (2–4) exciton transfer from A to B via dipole-dipole coupling, and (5) de-excitation of monomer B through a second interaction with the laser.

The appearance of the additional factor

$$J^2(G_{e,A} + G_{e,A,v,B})(G_{e,B} + G_{v,A,e,B})$$

is not incidental. It is precisely the same building block that appeared in the higher-order contributions to the diagonal matrix elements discussed in Sec. 1.1.4. The reason is identical: once the initial and final excitonic configurations are fixed, the two additional insertions of the interaction operator $\hat{V}$ are free to generate all intermediate states allowed by exciton exchange within the present minimal model.

Now, summing the all non-vanishing odd order perturbative terms leads to

$$\begin{aligned}\langle e_A, 0'_A; g_B, 0_B| G(\omega) |g_A, 0_A; e_B, 0'_B\rangle &= J G_{e,A} G_{e,B} \sum_{n=0}^{\infty} \Big[J^2(G_{e,A} + G_{e,A,v,B})(G_{e,B} + G_{v,A,e,B}) \Big]^n \\ &= \frac{J G_{e,A} G_{e,B}}{1 - J^2(G_{e,A} + G_{e,A,v,B})(G_{e,B} + G_{v,A,e,B})}. \end{aligned} \tag{S15}$$

Again, by symmetry,

$$\langle g_A, 0_A; e_B, 0'_B| G(\omega) |e_A, 0'_A; g_B, 0_B\rangle = \frac{J G_{e,A} G_{e,B}}{1 - J^2(G_{e,A} + G_{e,A,v,B})(G_{e,B} + G_{v,A,e,B})}. \tag{S16}$$

1.3 Final expression for the absorption spectrum of the dimer

Thus, the final expression for the absorption spectrum of the molecular dimer is

$$\begin{aligned}\langle g_A, 0_A; g_B, 0_B| \hat{\mu} \hat{G}(\omega) \hat{\mu} |g_A, 0_A; g_B, 0_B\rangle = &|\mu_M^A|^2 \langle e_A, 0'_A; g_B, 0_B| \hat{G}(\omega) |e_A, 0'_A; g_B, 0_B\rangle \\ &+ |\mu_M^A||\mu_M^B| \langle e_A, 0'_A; g_B, 0_B| \hat{G}(\omega) |g_A, 0_A; e_B, 0'_B\rangle \\ &+ |\mu_M^A||\mu_M^B| \langle g_A, 0_A; e_B, 0'_B| \hat{G}(\omega) |e_A, 0'_A; g_B, 0_B\rangle \\ &+ |\mu_M^B|^2 \langle g_A, 0_A; e_B, 0'_B| \hat{G}(\omega) |g_A, 0_A; e_B, 0'_B\rangle, \end{aligned} \tag{S17}$$

with

$$\langle e_A, 0'_A; g_B, 0_B|\hat{G}(\omega)|e_A, 0'_A; g_B, 0_B\rangle = \frac{G_{e,A}\Big[1 - J^2 G_{e,A,v,B}(G_{e,B} + G_{v,A,e,B})\Big]}{1 - J^2(G_{e,A} + G_{e,A,v,B})(G_{e,B} + G_{v,A,e,B})}, \tag{S18}$$

$$\langle g_A, 0_A; e_B, 0'_B|\hat{G}(\omega)|g_A, 0_A; e_B, 0'_B\rangle = \frac{G_{e,B}\Big[1 - J^2 G_{v,A,e,B}(G_{e,A} + G_{e,A,v,B})\Big]}{1 - J^2(G_{e,B} + G_{v,A,e,B})(G_{e,A} + G_{e,A,v,B})}, \tag{S19}$$

$$\langle e_A, 0'_A; g_B, 0_B|\hat{G}(\omega)|g_A, 0_A; e_B, 0'_B\rangle = \langle g_A, 0_A; e_B, 0'_B|\hat{G}(\omega)|e_A, 0'_A; g_B, 0_B\rangle = \frac{J G_{e,A} G_{e,B}}{1 - J^2(G_{e,A} + G_{e,A,v,B})(G_{e,B} + G_{v,A,e,B})}, \tag{S20}$$

where

$$G_{e,A} = \frac{1}{\omega - (\omega_{e,0,A} + \omega_{g,0,B}) + i\frac{\gamma_{e,A}}{2}}, \tag{S21}$$

$$G_{e,B} = \frac{1}{\omega - (\omega_{e,0,B} + \omega_{g,0,A}) + i\frac{\gamma_{e,B}}{2}}, \tag{S22}$$

$$G_{v,A,e,B} = \frac{1}{\omega - (\omega_{g,1,A} + \omega_{e,0,B}) + i\frac{\gamma_{v,A}+\gamma_{e,B}}{2}}, \tag{S23}$$

$$G_{e,A,v,B} = \frac{1}{\omega - (\omega_{e,0,A} + \omega_{g,1,B}) + i\frac{\gamma_{e,A}+\gamma_{v,B}}{2}}. \tag{S24}$$

2 Heterodimer simulation

We have derived exact expressions for the linear absorption spectra of a heterodimer in Section 1. Importantly, this framework can be straightforwardly extended to an arbitrary number of vibronic states on both the ground- and excited-state potential energy surfaces (PES). Consequently, one can compute the linear absorption spectrum of any heterodimer using this protocol, without any constraint on the value of J, i.e., the coupling need not be treated perturbatively. The only requirement is to parameterize the molecular dimer such that all parameters entering the Hamiltonian in Eq. 2 of the main text are determined: specifically, the energies and wavefunctions of the vibronic states, and the dipole–dipole coupling strength J appearing in Eq. 9 of the main text. Although the linear response expressions in Eq. S17 are exact, it is illuminating to explore certain perturbative regimes of this system, where J serves as the perturbative parameter, as we do below.

parameters	values in cm^{-1}	values in eV
$\omega_{e,0,A}$	14809	1.836
$\omega_{e,0,B}$	15037	1.864
$\omega_{g,1,A} - \omega_{g,0,A}$	350	4.3×10^{-2}
$\omega_{g,1,B} - \omega_{g,0,B}$	350	4.3×10^{-2}
J	52	6.5×10^{-3}
$\gamma_{e,A}, \gamma_{e,B}$	60	7.5×10^{-3}
$\gamma_{v,A}, \gamma_{v,B}$	2.4	3×10^{-4}

Table S1: Parameters of the Chlorophyll hetero dimer Chl522-Chl520 according to Ref. [7]

We consider a simulation with the parameters given in Table S1. These parameters correspond to the heterodimer studied in Ref. [7], where monomer A corresponds to Chl522 and monomer B corresponds to Chl520. Specifically, the site energies are taken from Table 3, the coupling J from Table 1, and the vibrational frequencies from Table 2 of Ref. [7]. Since Table 2 contains a large number of vibrational levels, we retain only a single representative mode in order to remain within the three-level system framework of the derived expressions in Eq. S17. The selected vibrational mode is among the strongest ones coupled to the electronic transition, as reflected by its Huang–Rhys factor in Table 2 of Ref. [7]. The electronic linewidth, modeled as a Lorentzian, is taken to be 60 cm^{-1}, as stated in the second paragraph below Eq. (7) of Ref. [7]. The vibrational linewidth is

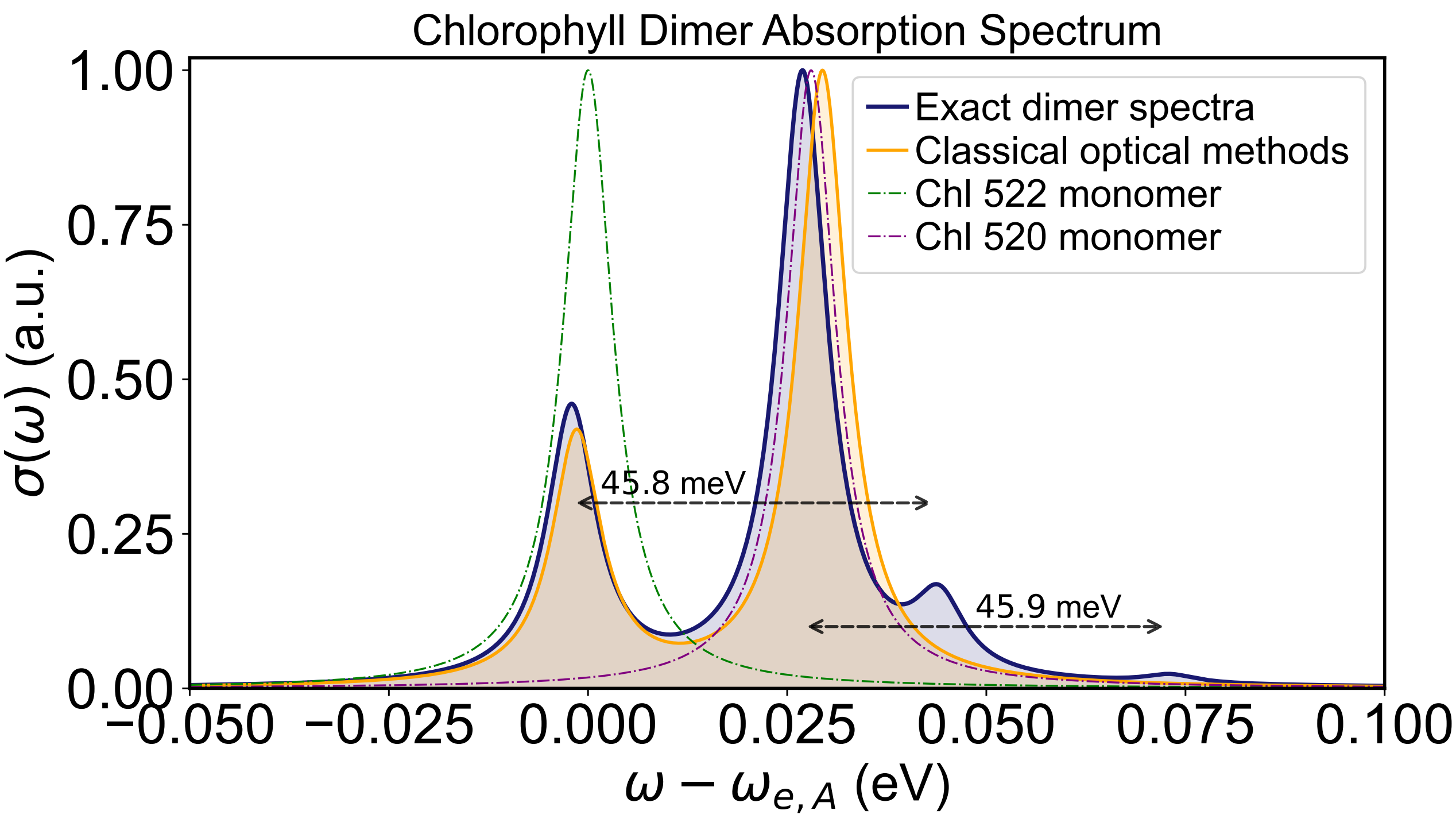

Figure S6: The absorption spectrum of the chlorophyll dimer (Chl522–Chl520), as shown in Fig. 1 of the main text, is reproduced here for reference.

taken to be 3×10^{-4} eV ([6]). Further, for simplicity, we have taken $|\mu_M^A| = |\mu_M^B| = 1$. The corresponding absorption spectrum is given in Fig. S6.

Here, the absorption peak of monomer A has an associated Raman (of monomer B) sideband that is shifted by approximately the vibrational gap $\omega_{\nu,B} = \omega_{g,1,B} - \omega_{g,0,B} = 0.043$ eV, and similarly, the absorption peak of monomer B has an associated Raman sideband shifted by approximately $\omega_{\nu,A} = \omega_{g,1,A} - \omega_{g,0,A} = 0.043$ eV. Thus, in this parameter regime, Raman-type information is directly encoded in the linear absorption spectrum of the heterodimer. This is consistent with the intuition developed from the ladder diagrams presented in Section 1: nonlinear information, specifically, the Raman contribution (which is part of the third-order susceptibility, $\chi^{(3)}(\omega)$), is embedded in the linear absorption spectrum of the molecular dimer.

Here, $\left|\frac{J}{\omega_{e,0,A}-\omega_{e,0,B}}\right| \approx 0.2$, placing the heterodimer in a regime where intermolecular coupling can be treated perturbatively. Thus, the spectra in Fig. 1 of the main text can be rationalized by expanding Eq. S17 in powers of J. Here, the Raman-type information appears in the diagonal components (see Section 1.1) at order J^2 (see Eq. S7), and in the off-diagonal components (see Section 1.2) at order J^3 (see Eq. S14). This is explicitly shown below:

$$\langle e_A, 0'_A; g_B, 0_B|\hat{G}(\omega)|e_A, 0'_A; g_B, 0_B\rangle = G_{e,A} + J^2 G_{e,A}^2 (G_{e,B} + G_{v,A,e,B}) + \mathcal{O}(J^4) \tag{S25}$$

$$\langle g_A, 0_A; e_B, 0'_B|\hat{G}(\omega)|g_A, 0_A; e_B, 0'_B\rangle = G_{e,B} + J^2 G_{e,B}^2 (G_{e,A} + G_{e,A,v,B}) + \mathcal{O}(J^4) \tag{S26}$$

$$\begin{aligned}\langle e_A, 0'_A; g_B, 0_B|\hat{G}(\omega)|g_A, 0_A; e_B, 0'_B\rangle = \langle g_A, 0_A; e_B, 0'_B|\hat{G}(\omega)|e_A, 0'_A; g_B, 0_B\rangle &= J G_{e,A} G_{e,B} \\ &+ J^3 G_{e,A} G_{e,B} (G_{e,A} + G_{e,A,v,B})(G_{e,B} + G_{v,A,e,B}) \\ &+ \mathcal{O}(J^5)\end{aligned} \tag{S27}$$

Thus, truncating the expansion consistently at $\mathcal{O}(J^2)$, the spectrum follows from Eq. S17. The diagonal Green's function elements are given by Eqs. S25 and S26, while the off-diagonal contribution (Eq. S27) enters only through its leading $\mathcal{O}(J)$ term,

$$\langle e_A, 0'_A; g_B, 0_B|\hat{G}(\omega)|g_A, 0_A; e_B, 0'_B\rangle = \langle g_A, 0_A; e_B, 0'_B|\hat{G}(\omega)|e_A, 0'_A; g_B, 0_B\rangle \tag{S28}$$

$$= J G_{e,A} G_{e,B}. \tag{S29}$$

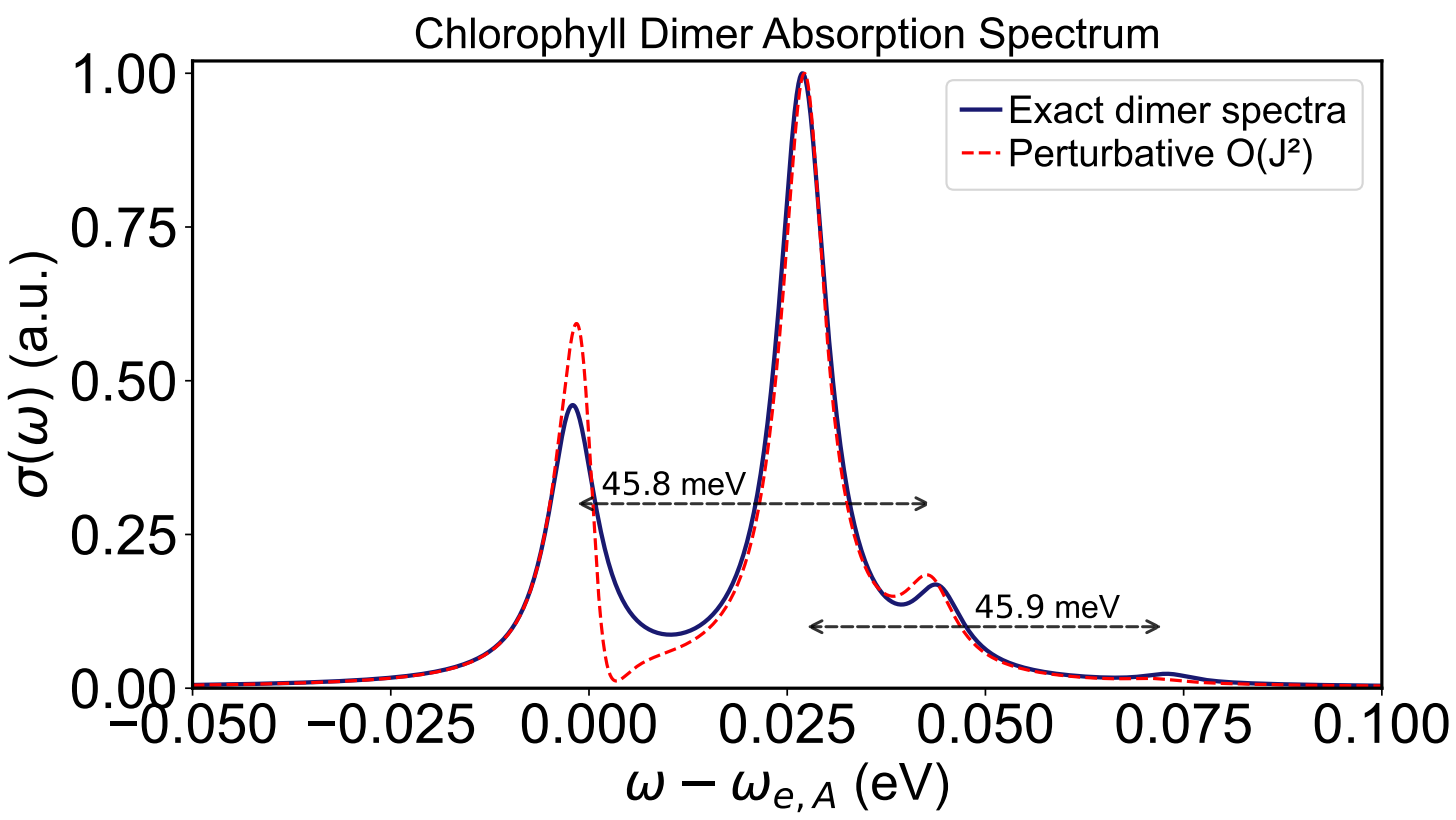

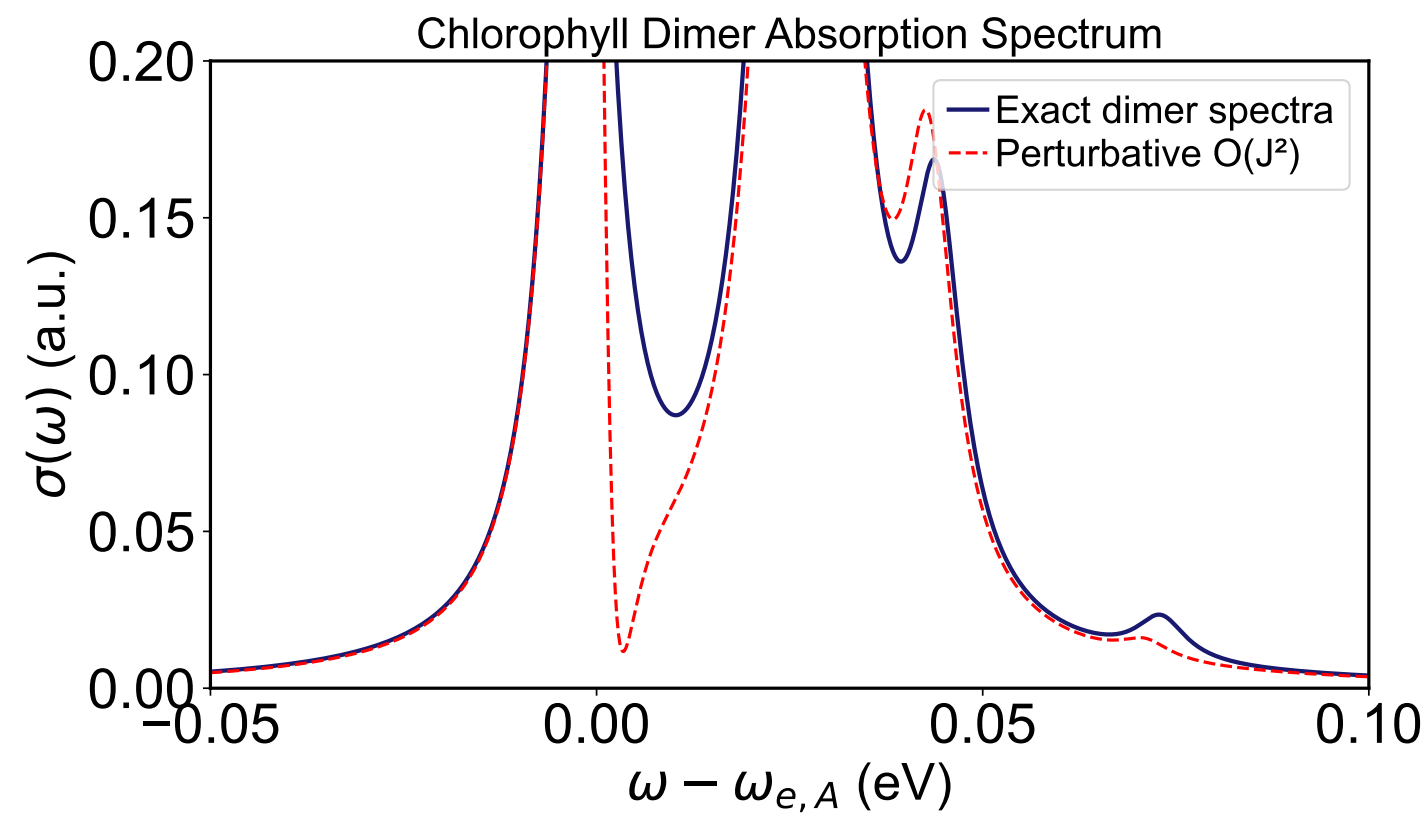

Figure S7: Perturbative analysis of the heterodimer spectra. The blue curve denotes the exact dimer spectrum, corresponding to Fig. 1 of the main text, while the red dashed curve is obtained by retaining terms only through $\mathcal{O}(J^2)$ in Eq. (S17). As shown in the left panel, this perturbative correction is sufficient to reproduce the Raman feature near $\omega - \omega_{e,A} \approx 0.05$. The right panel, which provides a zoomed-in view of the left panel, further shows that the same $\mathcal{O}(J^2)$ approximation captures the Raman feature near $\omega - \omega_{e,A} \approx 0.075$.

The diagonal components contain zeroth-order terms $G_{e,A}$ and $G_{e,B}$ (see Eq. S25 and S26), which reproduce the absorption features of the isolated monomers A and B, respectively. The off-diagonal terms, which are first order in J, contribute additional oscillator strengths at the electronic transition frequencies of both monomers, as can be seen from the poles of $JG_{e,A}G_{e,B}$.

Turning now to the central result, we examine the $\mathcal{O}(J^2)$ terms: $J^2G_{e,A}^2(G_{e,B} + G_{v,A,e,B})$ and $J^2G_{e,B}^2(G_{e,A} + G_{e,A,v,B})$. The first parts of these expressions, $J^2G_{e,A}^2G_{e,B}$ and $J^2G_{e,A}G_{e,B}^2$, contribute poles at the bare electronic transition frequencies, thereby adding oscillator strength around the monomer peaks (also introducing some shifts) in this perturbative regime. The second parts, $J^2G_{e,A}^2G_{v,A,e,B}$ and $J^2G_{e,B}^2G_{e,A,v,B}$, are more significant: they generate poles shifted by the vibrational energy gaps of the coupled (other) monomer. Specifically, $J^2G_{e,A}^2G_{v,A,e,B}$ creates a pole at $\omega = \omega_{e,0,B} + (\omega_{g,1,A} - \omega_{g,0,A})$, as seen in Fig. S6, and similarly, $J^2G_{e,B}^2G_{e,A,v,B}$ creates a pole at $\omega = \omega_{e,0,A} + (\omega_{g,1,B} - \omega_{g,0,B})$, also seen in Fig. S6. These perturbative analyses are shown in Fig. S7 for reference. Extending this analysis, the entire Raman spectrum of the embedded monomers can in principle be read off from the linear absorption spectrum of the heterodimer, in the spirit of Ref. [6].

Finally, we note that in Fig. S6, the Raman sideband features are not shifted by exactly the vibrational frequency; there are additional small shifts present. We attribute these to higher-order contributions of $\mathcal{O}(J^4)$ not retained in our perturbative analysis, which can be systematically computed for any system of interest.

Further, the asymmetry in the absorption peak heights (compared to the uncoupled monomer peak heights in Fig. S6) is a direct fingerprint of the *sign* of the coupling strength J. Since J is positive, even its perturbative influence is sufficient to imprint a bright–dark character onto the two eigenstates of the dimer, a phenomenon similar to what is observed in molecular H- and J-aggregates. To see why, recall that the dipole–field interaction (the $\hat{\boldsymbol{\mu}} \cdot \mathbf{E}$ term embedded in Eq. 8 of the main text) selectively drives transitions to states whose transition dipole moments add constructively. When $J > 0$, it is the higher-energy eigenstate that acquires the dominant bright character. This superposition, in which the individual monomer transition dipoles combine *in-phase*, leads to increased oscillator strength near the electronic transition frequency of monomer B. The lower-energy eigenstate, by contrast, receives a comparatively smaller component of the total oscillator strength, which is why the absorption feature near the transition frequency of monomer A appears diminished. This logic inverts cleanly when $J < 0$: the bright and dark characters exchange roles, the oscillator strength migrates to the lower-energy eigenstate, and the absorption peak near the transition frequency of monomer A would instead dominate.

In summary, we have established a protocol by which the linear absorption spectrum of a heterodimer directly encodes the Raman features of its constituent monomers.

3 Linear response of linear molecular aggregates

In this section, we discuss the calculations performed on the linear response of linear molecular aggregates that is presented in the manuscript in Fig. (2). In order to obtain the difference between the CPA features and the Raman features, we have

Parameters	value
λ^2	1
ω_v	1 eV
J	-3 eV
γ	0.18 eV
N	10
M	2

Table S2: Parameters corresponding to the linear absorption spectra of linear aggregates shown in Fig. (2) of the manuscript. Here, $M = 2$ is the number of vibrational states considered in ground electronic potential energy surface.

compared the CPA spectra for the linearly coupled molecular aggregate system with the parameters shown in Table (S2) with the exact spectra computed for aggregates made up of $N = 10$ monomers. Further, the vibrational degrees of freedom of the molecules are modeled through shifted harmonic oscillator model [1], leading to the following Hamiltonian,

$$H_e = \omega_v \sum_n b_n^\dagger b_n + \omega_v \lambda \sum_n (b_n^\dagger + b_n) |n\rangle \langle n| + \sum_n J\Big(|n+1\rangle \langle n| + |n\rangle \langle n+1| \Big) + \omega_e + \lambda^2 \omega_v,$$

where we have considered the monomers to be interacting only with its nearest neighbors. Here, ω_e is the monomer electronic excitation energy with λ^2 being the Huang-Rhys factor modeling the vibronic coupling with the harmonic vibration characterized by ω_v. Further, b_n is the annihilation operator of the vibrational mode and $|n\rangle$ is the state corresponding to the electronic excitation only in the nth monomer. Finally, the linear absorption spectra of this system is computed using

$$\sigma(\omega) \propto -\Im \langle G| \mu G(\omega) \mu |G\rangle ,$$

where $G(\omega)$ is the Green's function of the whole system, μ is the total dipole moment operator and $|G\rangle$ represents the state of the system where all the monomers are in the global ground state.

4 Many particle approximations

In addition to Fig. (2) shown in the manuscript, where we interpret the corrections to the CPA in the molecular aggregate spectra as Raman signatures, we also explore in this section the features that are captured by the Two-Particle Approximation (TPA). In Fig. (S8), we show the exact simulation of the nearest-neighbor–coupled molecular aggregate system explored in the literature (Fig. 17(i) in Hestand et al. [1]). We observe Raman-type signatures appearing in the strongly coupled regime, similar to Fig. (2) in the manuscript, which are captured by the TPA. Although the exact spectra capture finer features than the TPA, the Raman-type features already appear within the TPA. This is not entirely surprising, as the two-particle states in the TPA include configurations where monomers host vibrational excitations in the ground electronic state.

Although the TPA captures these Raman-type features, and similar features have been reported previously, they were not identified as Raman-type in origin. Consequently, it was not recognized that the associated spectral shifts encode Raman-type information. We believe that this constitutes one of the key contributions of our work: identifying a previously unrecognized spectroscopic insight that can be recovered within approximations already well established in the molecular-aggregate community. More broadly, our result reveals a quantum-optical effect through which nonlinear Raman-type information about individual monomers can be accessed from the linear response of an aggregate.

References

[1] Nicholas J Hestand and Frank C Spano. Expanded theory of h-and j-molecular aggregates: the effects of vibronic coupling and intermolecular charge transfer. *Chemical reviews*, 118(15):7069–7163, 2018.

[2] Arghadip Koner and Joel Yuen-Zhou. Hidden nonlinear optical susceptibilities in linear polaritonic spectra. *Optica*, 12(10):1625–1631, 2025.

[3] S. Mukamel. *Principles of nonlinear optical spectroscopy.* Oxford University Press, 1995.

[4] Juan B Pérez-Sánchez, Arghadip Koner, Sricharan Raghavan-Chitra, and Joel Yuen-Zhou. Cut-e as a 1/n expansion for multiscale molecular polariton dynamics. *The Journal of Chemical Physics*, 162(6), 2025.

Parameters	Value
N	6
ω_0	1eV
λ^2	1
$J_{n,n+1}$	-1eV
M	2
γ	0.18eV

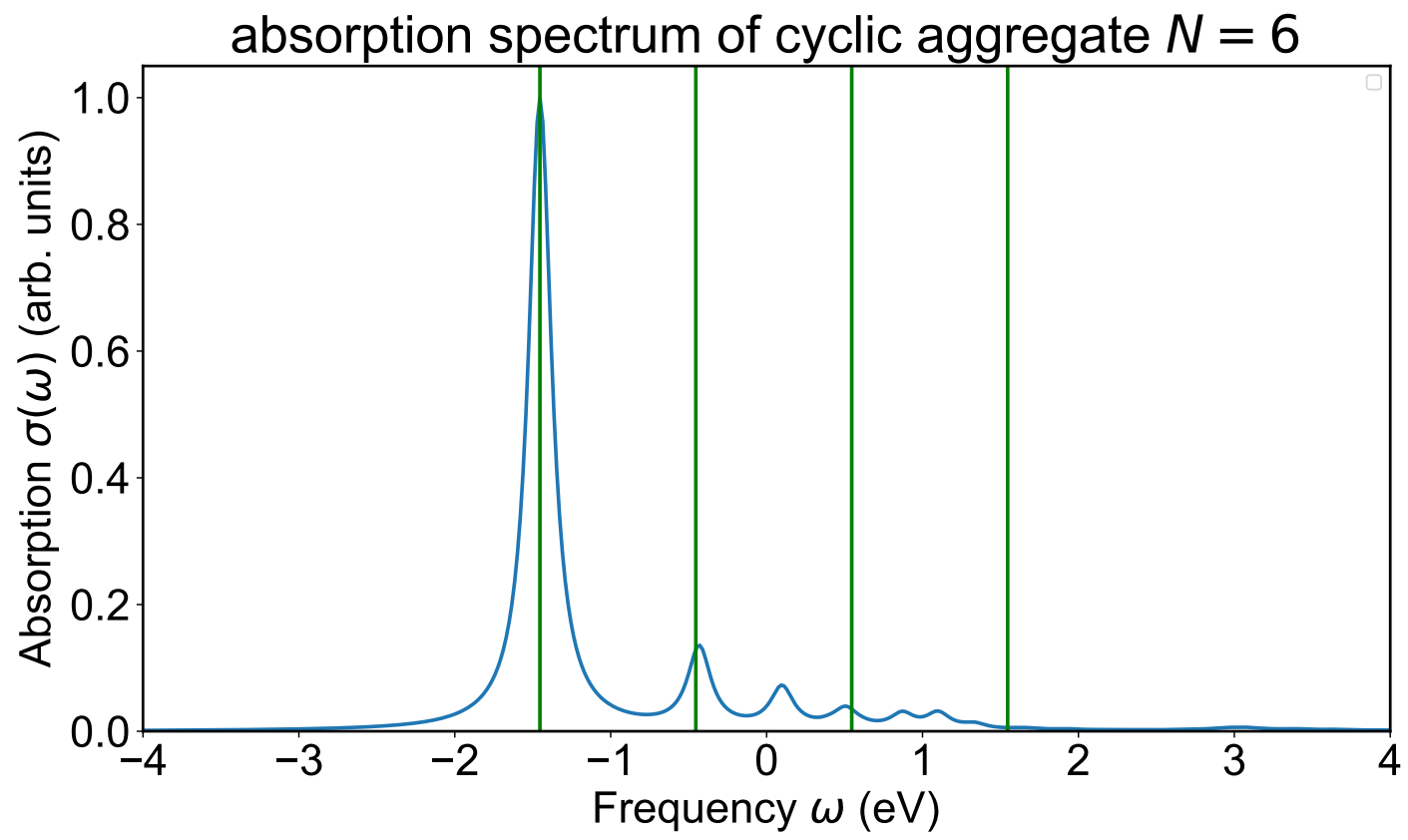

Figure S8: Parameters and the corresponding exact absorption spectra for an idealized circular aggregates, where all of the dipole moments are aligned. The parameters correspond to the Fig. 17 (i) in Hestand et al. [1]. We have used eV for the purpose of the simulation, however, the unit doesn't affect the spectra. Here, $M = 2$ is the number of vibrational states considered in ground electronic potential energy surface.

[5] Juan B Pérez-Sánchez, Arghadip Koner, Nathaniel P Stern, and Joel Yuen-Zhou. Simulating molecular polaritons in the collective regime using few-molecule models. *Proceedings of the National Academy of Sciences*, 120(15):e2219223120, 2023.

[6] Sricharan Raghavan-Chitra, Arghadip Koner, and Joel Yuen-Zhou. High-q microresonators unveil quantum rare events. *arXiv preprint arXiv:2510.27034*, 2025.

[7] Mike Reppert, Khem Acharya, Bhanu Neupane, and Ryszard Jankowiak. Lowest electronic states of the cp47 antenna protein complex of photosystem ii: simulation of optical spectra and revised structural assignments. *The Journal of Physical Chemistry B*, 114(36):11884–11898, 2010.

[8] Andrei Tokmakoff. Nonlinear and two-dimensional spectroscopy notes. Accessed: 04-20-2025.
